# Stability of Treatment Effects and Caregiver-Reported Outcomes: A Meta-Analysis of Trauma-Focused Cognitive Behavioral Therapy for Children and Adolescents

**DOI:** 10.1177/10775595231167383

**Published:** 2023-03-23

**Authors:** Jonathan Felix Benjamin Thielemann, Barbara Kasparik, Julia König, Johanna Unterhitzenberger, Rita Rosner

**Affiliations:** 1 Department of Psychology, 26541 Catholic University of Eichstätt-Ingolstadt, Ingolstadt, Germany; 2 Alexianer Krefeld GmbH, Hospital Maria-Hilf, Clinic for Mental Health, Department of General Psychiatry, Krefeld, Germany; 3 Centre for Children and Adolescents Inn-Salzach e.V., Department of Child and Adolescent Psychiatry, Altoetting, Germany

**Keywords:** adolescents, caregivers, child, meta-analysis, traumatic stress, longterm effects

## Abstract

The efficacy of trauma-focused treatments for children and adolescents is well researched. However, less is known about the long-term and caregiver-reported effects. Searched databases were PsychInfo, MEDLINE, Cochrane Library, PTSDPubs, PubMed, Web of Science, and OpenGrey. Treatment effects of trauma-focused cognitive behavioral therapy (TF-CBT) were computed at 12-month follow-up with posttraumatic stress symptoms (PTSS) as primary outcome and symptoms of depression, anxiety, and grief as secondary outcomes. Concordance between participant and caregiver ratings were investigated. TF-CBT showed large improvements across all outcomes from pre-treatment to 12-month follow-up (PTSS: g = 1.71, CI 1.27–2.15) and favorable results compared to active treatments and treatment as usual at 12-month follow-up (PTSS: g = .35, CI .13–.56). More pronounced effects were found in group settings. No significant differences were detected between participant and caregiver ratings with high reliability across almost all outcomes and assessment points. TF-CBT is a reliable treatment for pediatric PTSS and secondary symptoms with stable results at 12-month follow-up.

## Introduction

Rates of traumatic experiences are high among children and adolescents with one US study finding that half their sample had experienced a traumatic event in the last year (Finkelhor et al., 2013). In children exposed to traumatic events, a conditional prevalence rate of 15.9% is estimated for posttraumatic stress disorder (PTSD; Alisic et al., 2014). Symptoms of depression and anxiety are common concomitants (American Psychiatric Association, 2013) with research suggesting comorbid diagnoses in half of the pediatric PTSD cases (Kar & Bastia, 2006). According to international guidelines, trauma-focused cognitive behavioral therapy (TF-CBT) is the treatment of choice for pediatric PTSD with caregiver participation being an important treatment factor (Forbes et al., 2020; National Institute for Health and Care Excellence, 2018; Phoenix Australia Centre for Posttraumatic Mental Health, 2013).

In the pediatric TF-CBT literature, TF-CBT is used as a generic term for CBT with trauma-focused work as well as specifically for the manual of Cohen et al. (2006, 2017) In order to make a clear distinction, the latter will be referred to as ‘specific TF-CBT’. In the context of pediatric TF-CBT treatments, specific TF-CBT is the most widely evaluated treatment protocol. It includes standard cognitive behavioral therapy (CBT) techniques, forming the acronym PRACTICE: psychoeducation and parenting skills (P), relaxation (R), affective modulation (A), cognitive coping (C), trauma narrative (T), in vivo exposure (I) conjoint parent-child sessions (C) and enhancing safety and development (E). According to the developers, at least eight sessions are necessary to cover all components with 45 minutes each assigned to the child and caregiver. Hence, caregiver involvement is essential in specific TF-CBT. For a more extensive overview, the reader is referred to our previous review and meta-analysis (Thielemann et al., 2022).

Although specific TF-CBT has been previously confirmed as an effective treatment (Cary & McMillen, 2012; Thielemann et al., 2022), little is known about the stability of treatment effects and the agreement between children and adolescents (self-report) and their caregivers (caregiver-report) regarding youths’ symptoms in the context of specific TF-CBT. While the effectiveness of interventions is an important outcome, we also have to consider whether these effects can be sustained over time to choose treatments that achieve the best long-term outcomes for patients as well as the healthcare system. Furthermore, if we continue to use caregiver-reports as an outcome for pediatric posttraumatic stress symptoms (PTSS) and secondary symptoms in TF-CBT studies, we need to assess whether they reflect children and adolescents’ experience or provide different information. Should the assessment not correspond, we also need to investigate the degree and direction of disagreement to understand their relation and their individual value.

### Summary of Previous Analyses

As mentioned above, in the pediatric TF-CBT literature, TF-CBT is used as a generic term as well as specifically for the specific TF-CBT manual. Accordingly, next to the specific TF-CBT manual, most reviews and meta-analyses on TF-CBT included other trauma-focused CBT manuals such as EMDR (Shapiro, 2018), CBITS (Jaycox, 2018), KIDNET (Neuner et al., 2008), PE (Foa et al., 2019) and CPT (Resick et al., 2017) among several others (Hoogsteder, Thije, Schippers, & Stams, 2021; Lenz & Hollenbaugh, 2015; Lewey et al., 2018; Mavranezouli et al., 2020; Morina et al., 2016). Additionally, most of them did not include follow-up assessments (Hoogsteder et al., 2021; Lenz & Hollenbaugh, 2015; Lewey et al., 2018) or merged all follow-up assessment points covering different periods (Mavranezouli et al., 2020; Morina et al., 2016) and none considered concordance between self-reported and caregiver-reported outcomes.

The first systematic review on specific TF-CBT found positive small to medium effect sizes compared to active non-CBT control conditions for PTSS and depression at post-treatment (Cary & McMillen, 2012). At 12-month follow-up, the effect for PTSS was maintained but depression only yielded a small non-significant effect. The authors found the same pattern for variants of TF-CBT that did not strictly adhere to the manual. Unfortunately, studies with CBT control groups were excluded from analyses, limiting the analyses on specific TF-CBT to three randomized controlled trials (RCTs) at the time.

Apart from that, only two other meta-analyses on TF-CBT addressed follow-up assessments (Mavranezouli et al., 2020; Morina et al., 2016). However, they did not conduct analyses for specific TF-CBT but included other manuals in their analyses (e.g. CPT, KIDNET, PE, CBITS). Mavranezouli et al. (2020) found a large positive post-treatment effect on PTSS compared to wait-list conditions. At 1–4-month follow-up, the effect was not only sustained but the large effect size increased further. In contrast, Morina et al. (2016) found a small positive post-treatment effect on PTSS compared to active treatments that disappeared at 3–24-month follow-up. However, these findings are difficult to compare due to the different follow-up periods and comparators used. Additionally, Morina et al. (2016) analyzed depression at 3–24-month follow-up. However, effect sizes could neither be calculated for wait-list nor active treatments but only for active control conditions containing psychoeducation, supportive counselling and treatment as usual. Compared to these control conditions, TF-CBT maintained a medium effect on depression.

To the best of our knowledge, only two meta-analyses have attempted to investigate concordance between self-reported and caregiver-reported symptoms in children and adolescents (Achenbach et al., 1987; Los Reyes et al., 2015). Both of them found low to moderate concordance between raters with somewhat greater agreement for externalizing than internalizing symptoms. This is mostly likely due to the subjective experience of internalizing symptoms that is directly only accessible by the individual (Asbrand et al., 2021; Los Reyes et al., 2015). Thus, for caregivers, they are more difficult to observe than externalizing symptoms. Additionally, children and adolescents with more internalizing symptoms might have been more withdrawn and interacted less with their caregivers, providing fewer situation for caregivers to recognize their problems (Bass et al., 2014). Concerning PTSS, most studies that have investigated concordance between children and adolescents and their caregivers also showed limited concordance with caregivers reporting a lower symptom load (Exenberger et al., 2019; Meiser-Stedman et al., 2007; Scheeringa et al., 2006; Schreier et al., 2005; Shemesh et al., 2005; Stover et al., 2010). However, some studies indicate that the reports tend to converge over time (Meiser-Stedman et al., 2007; Schreier et al., 2005). While discrepancies between reporters were often discussed in terms of measurement error, they can also be considered as the unique perspectives of different observers and the context-specific symptom occurrence (Los Reyes et al., 2015). That is, some behaviors may only be observable in a specific context (e.g. with peers) or only recognized by children and adolescents or their caregivers. In this sense, discrepancies are different yet valid information that can greatly assist diagnosis and treatment decisions. Interestingly, some studies from populations with physical illnesses such as cancer (Clawson et al., 2013; Erickson et al., 2017; Phipps et al., 2005) and epilepsy (Stevanovic et al., 2012) found significant moderate to high correlations between self-reported and caregiver-reported PTSS, depression and anxiety as well as no mean difference between raters. This contrary finding may be explained by a greater awareness and involvement of parents in their children’s health and treatment in this population. As parallel sessions with caregivers are an important component of specific TF-CBT, this effect may also be present. However, concordance between children and adolescents and their caregivers regarding youths’ symptoms in the context of specific TF-CBT has not been investigated by meta-analysis yet.

### Current Study

Our previous analysis confirmed the ability of specific TF-CBT to reduce PTSS and comorbid symptoms, as well as its superiority to other treatment approaches (Thielemann et al., 2022). These results were in line with earlier findings on specific TF-CBT (Cary & McMillen, 2012; Gutermann et al., 2016) and the broader TF-CBT literature (Lenz & Hollenbaugh, 2015; Mavranezouli et al., 2020; Morina et al., 2016). In contrast to previous analyses (Gutermann et al., 2016; Singal et al., 2014), we also found greater effects on PTSS in group settings and effectiveness trials. However, we only assessed symptoms post-treatment and restricted outcomes to self-report and clinical interviews. Thus, it is still unclear how stable these effects are and how well the reported symptoms concur with the experience of the caregivers in specific TF-CBT who interact with the children and adolescents on a day-to-day basis and are an integral part of the treatment. In light of these results and the very few meta-analyses on follow-up periods as well as the absence of meta-analyses of concordance between self-reported and caregiver-reported pediatric PTSS and secondary outcomes in the context of specific TF-CBT, an investigation into these issues is warranted. This meta-analysis quantifies the treatment effects of specific TF-CBT from pretreatment to 3-month follow-up (FU I), 6-month follow-up (FU II) and 12-month follow-up (FU III), from FU II to FU III as well as in comparison to control conditions at the follow-up assessment points for PTSS and secondary outcomes of depression, anxiety and grief. FU III was defined as the primary endpoint as the other follow-ups were assessed relatively early after treatment and we expected fewer studies to be included in these analyses. Nevertheless, we included the earlier follow-up periods to cover as many studies as possible and to have a closer look at the course of symptoms over time. RCTs, individual and group settings as well as effectiveness and efficacy trials will be considered separately for the primary endpoint.

## Methods

### Search and Screening of Studies

**Figure 1.**
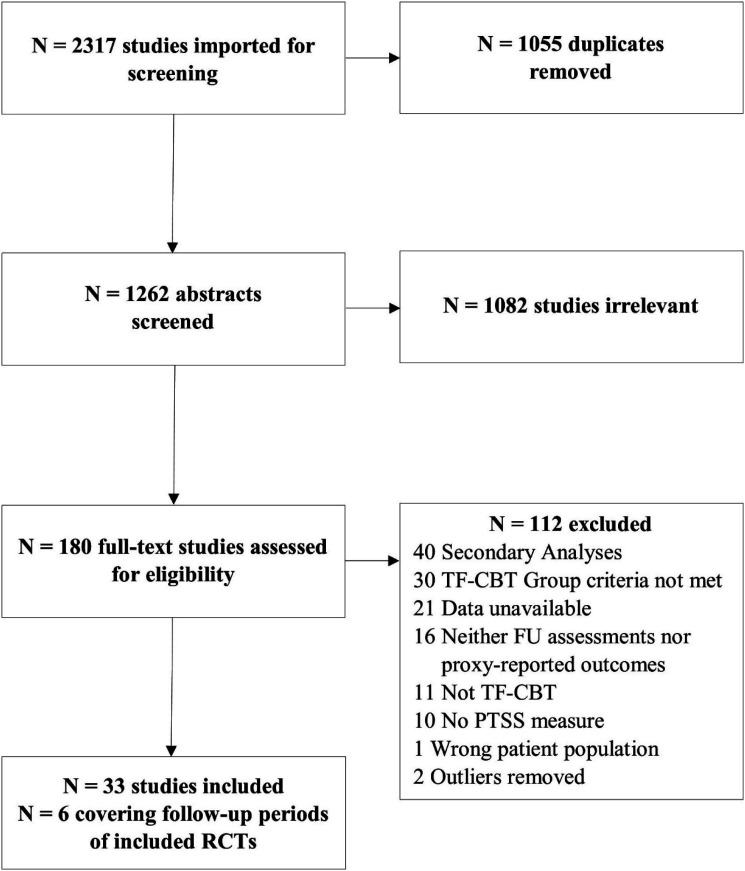
Flowchart study selection. *Note.* The two outliers refer to the same study covering the follow-up period.

To ensure comparability, this meta-analysis used the same search terms as in our previous analysis (Thielemann et al., 2022; PROSPERO: CRD42020139403). Databases included PsychInfo, MEDLINE, Cochrane Library, PTSDPubs, PubMed and Web of Science as well as OpenGrey and were searched with a pre-defined combination of search terms for articles published between Jan 1^st^, 1990 to Aug 19^th^, 2021 (see Table 1). In addition, we conducted a manual search of reference sections of relevant works and sought expert suggestions, resulting in 1262 publications without duplicates (see Figure 1). No language limitations were applied. Covidence (Veritas Health Innovation, 2014) was used for title and abstract screening with two independent raters (JT and BK) assessing the articles. Conflicting assessments were solved in discussion by reviewing the abstracts. Full-text readings and assessments of inclusion and exclusion criteria were conducted by the first author. If assessments were inconclusive, authors were contacted and articles were discussed with the co-authors to resolve the issues.

**Table 1.** Pre-Defined Search Terms.

Search Categories	Search Terms
Diagnosis	Trauma* or posttrauma* or post-trauma* or PTSD or PTSS or grief or griev*
Trauma-related	Abuse* or assault* or abduct* or accident* or kidnapp* or life-threat* or maltreat* or mistreat* or neglect* or refugee or shooting or terroris* or victim* or violence or war or hurricane or tsunami or earthquake or flood or “natural disaster” or bereave* or loss
Youth	Adolescen* or child* or youth or kid or juvenile or infant or minor or teenager or young*
TF-CBT	“Trauma focused cognitive behavioral treatment” or “trauma-focused cognitive behavioral treatment” or “trauma focused cognitive behavioral therapy” or “trauma-focused cognitive behavioral therapy” or “trauma focused cognitive behavior*” or “trauma-focused cognitive behavior*” or “trauma focused cog*” or “trauma-focused cog*” or “trauma focused” or trauma-focused or TF-CBT or grief-focused or “grief focused”

*Note.* Combination: (Diagnosis or Trauma-related) and Youth and TF-CBT.

### Inclusion and Exclusion Criteria

The following inclusion criteria were originally applied to TF-CBT studies conducted in individual settings: (1) age range of 3–21 years, (2) participants had been exposed to at least one traumatic experience, and (3) had to complete at least 8 sessions (4) of specific TF-CBT (Cohen et al., 2006; 2017) or earlier versions of the same manual (Cohen & Mannarino, 1993; Deblinger & Heflin, 1996). Additionally, (5) results on PTSS were assessed pre and post-treatment with a quantitative self-report measure or clinical interview. (6) Moreover, only original research was considered, excluding reviews, meta-analyses, and case reports. For the present meta-analysis, we added an additional criterion: Either (7a) studies had to include at least one follow-up assessment of PTSS, depression, anxiety or grief reported via self-report or clinical interview, or (7b) include caregiver-reported outcome measure for PTSS, depression, anxiety or grief assessed at least pre and post-treatment. The only exclusion criterion was if children did not receive treatment (e.g. only parents were treated). If PTSS pre-post effect sizes could not be extracted (c.f. criterion 5), we approached the authors and included the article if supplementary data was supplied.

The inclusion criteria for TF-CBT studies conducted in group settings differed only slightly: no minimum number of sessions was required, and treatment did not need to include all PRACTICE components. Instead, it needed to contain (1) psychoeducation, (2) coping strategies (i.e. relaxation, affective modulation, cognitive coping), (3) exposure, (4) cognitive processing/restructuring of trauma-related thoughts and beliefs and (5) some reference to the manual or its earlier versions.

### Treatment and Control Groups

Control groups consisted of randomized wait list, treatment as usual (TAU) and active treatment (AT) conditions. TAU and AT were merged as interventions were comparable. If two TAU/AT control groups were reported, we used the higher treatment dose. If two TF-CBT conditions were reported, we included them both in the pre-post analysis unless results were merged in the original report. If a control group was reported as well, it was compared to both TF-CBT conditions, separately. One study used a combination of four TF-CBT conditions and four control groups that were comparable regarding the treatment dose (Dorsey et al., 2020). These control groups were paired with the corresponding TF-CBT condition as data collection was conducted in different countries and regions.

### Efficacy and Effectiveness

Efficacy trials take place in highly controlled settings to increase internal validity while in effectiveness trials, treatments are implemented directly in the health care system (Singal et al., 2014). We distinguished the two by adapting the classification criteria from Gartlehner et al. (2006) considering study title, settings, inclusion criteria, analyses, adaptations, therapists, caregiver participation, as well as control conditions. The detailed criteria can be found in S6. The first and second authors (JT and BK) independently assessed all studies and solved inconsistent ratings in discussion. However, an unequivocal distinction is sometimes difficult as efficacy and effectiveness trials function on a continuum and may include some characteristics of the other trial type. Decisions were made depending on which criteria preponderated.

### Risk of Bias Assessment

The first and second author (JT and BK) assessed risk of bias with the Risk of Bias assessment tool (RoB 2.0; Sterne et al., 2019) and the Risk Of Bias In Non-randomized Studies – of Interventions (ROBINS-I) assessment tool (Sterne et al., 2016), resulting in ‘low risk’, ‘some concerns’ or ‘high risk’ ratings. The tools present five (Rob 2.0) to seven (ROBINS-I) domains to assess risk of bias. For the latter, only four domains were used since the concerns of the other domains were covered by the inclusion criteria and we did not include non-randomized control groups. Four RCTs and seven uncontrolled studies were identified as ‘high risk’ studies and excluded from analyses. For a more detailed overview, the reader is referred to our previous meta-analysis (Thielemann et al., 2022).

### Outcomes and Data Extraction

All data were extracted by the first and second authors including outcome data on PTSS, depression, anxiety, and grief as well as caregiver-reported outcomes on PTSS, depression, anxiety and grief. Clinical interviews were the first choice of participants’ data. If unavailable, we used self-report instruments instead. When appropriate, we merged subscales using the Cochrane formula (Higgins et al., 2022). We contacted authors for missing information and excluded studies from the originally selected sample (Thielemann et al., 2022) if no outcome data could be acquired for follow-up or pre- to post-treatment caregiver-reported outcomes.

#### Statistical Analyses

All eligible studies were included in respective analyses generating effect sizes (Hedges’ g and 95% CIs) for PTSS, depression, anxiety and grief. We used Comprehensive Meta-Analysis, version 3 (Borenstein et al., 2013) for meta-analyses and IBM SPSS Statistics (Version 25) for computing two-way mixed method absolute agreement intra class coefficients and t-tests (α = .05). If completer data and intention-to-treat data were reported, the latter were our first choice. If necessary, we imputed correlations for pre-post effect sizes based on the overall mean of available correlations for the respective outcome from our first meta-analysis and assumed the same for later assessment points. As we were able to include fewer studies than in our original meta-analysis, we also calculated pre-post (within-group) and post (between-group) effect sizes to ensure effects were comparable to our previous results. We assumed a random-effects model as there was a lot of heterogeneity in the samples (Hedges & Vevea, 1998) and confirmed it with Q statistic (Higgins et al., 2003). When Q was non-significant, we accepted a fixed model, and reported effect sizes accordingly. I^2^ was used to estimate heterogeneity. For the detection of outliers (Hedges’ g > 4), we used funnel plots. We performed additional subgroup analyses when at least three TF-CBT conditions or three post-treatment comparisons were available and the respective counterpart (individual and group; efficacy and effectiveness) could be calculated as well. We detected publication bias in some analyses and addressed it with Duval and Tweedie’s (2000) trim and fill method. We computed intraclass correlations between self-reported and caregiver-reported outcomes at the different assessment points and compared ratings using t-tests.

## Results

Study selection is shown in Figure 1. An overview of all included studies can be found in the Supplementary Material S1. We ultimately selected 33 studies of which 21 were RCTs and 12 were uncontrolled studies. Most studies used an individual treatment setting (*k* = 26, 15 of them RCTs) and some were conducted in a group setting (*k* = 7, six of them RCTs). Of the RCTs, 11 were efficacy trials and 10 were effectiveness trials. While 20 studies (*k* = 16 RCTs) were considered for follow-up analyses of self-reported outcomes, 21 studies (*k* = 9 RCTs) were included in the concordance analyses of self-reported and caregiver-reported outcomes. Wait list control conditions were included in six RCTs and TAU/AT control conditions in 11 RCTs. In three RCTs, the control condition was a second TF-CBT condition, which we used as an additional treatment condition. In the remaining RCT, the control condition was excluded as some participants received TF-CBT while others did not. Thus, it represented neither a viable control groups nor a clear-cut TF-CBT condition. The within-group and between-group effect sizes for the primary endpoint (FU III) can be found in Table 2 and Table 3, respectively. High risk of bias studies were excluded from all analyses if not otherwise noted. Table 4 shows the effect sizes for caregiver-reported outcomes. Means, SDs, intraclass correlations and t-statistics for self-reported and caregiver-reported outcomes can be found in Table 5. For the effect sizes of individual studies and the funnel plots showing observed and imputed studies, see supplementary material S2. Uncontrolled effect sizes for intermediate follow-up analyses (pre to FU I and FU II) as well as uncontrolled pre-post and controlled post-treatment effect sizes for the respective studies involved in the follow-up analyses can be found in S3. Risk of bias assessments and analyses including high risk studies can be found in S4 and S5, respectively.

**Table 2.** Pre-FU III and FU II-FU III Within-Group Effect Sizes for PTSS, Depression, Anxiety and Grief.

Assessment Points	Outcome	Sample		*n*	g	95% CI	Se	z	Q	I^2^	Duval and Tweedie
Pre-FU III	PTSS	All studies		13	(1.98) 1.71^a^	(1.53–2.43) 1.27–2.15	(.23)	(8.66***)	117.65***	89.80	3
		Individual		8	1.63	1.07–2.19	.29	5.67***	61.76***	88.67	0
		Group		5	2.47^b^	2.25–2.69	.11	21.81***	6.87	41.79	0
		RCTs only		12	(1.93) 1.71^a^	(1.46–2.39) 1.24–2.18	(.24)	(8.08***)	109.86***	89.99	2
			Efficacy	6	1.69	.80–2.57	.46	3.74***	60.95***	91.80	0
			Effectiveness	6	(2.16) 2.00^a^	(1.69–2.62) 1.53–2.47	(.24)	(9.10***)	31.86***	84.31	1
			Individual	8	1.63	1.07–2.19	.29	5.67***	61.76***	88.67	0
			Group	4	2.44^b^	2.20–2.68	.12	19.91***	6.41	53.18	0
	Depression	All studies		9	.99	.70–1.29	.15	6.55***	37.48***	78.65	0
		RCTs only		8	.90	.63–1.17	.14	6.46***	23.86**	70.66	0
	Anxiety	All studies		6	.95	.55–1.35	.20	4.69***	29.84***	83.25	0
		RCTs only		6	.95	.55–1.35	.20	4.69***	29.84***	83.25	0
	Grief	All studies		6	(1.73) 1.44^a^	(1.29–2.18) .94–1.94	(.23)	(7.62***)	28.67***	82.56	2

		RCTs only		5	(1.61) 1.44^a^	(1.12–2.10) .91–1.97	(.25)	(6.47***)	24.24***	83.50	1

FU II-FU III	PTSS	All studies		7	.20^b^	.07–.33	.07	3.05**	1.68	0	0
	Depression	All studies		7	.09^b^	−.04–.21	.06	1.37	3.25	0	0
	Anxiety	All studies		5	.12	−.02–.26	.07	1.70	3.65	0	0

*Note.* High risk of bias studies are excluded; *n* = number of included TF-CBT conditions. *p < .05; **p < .01; ***p < .001.

^a^Recalculated with imputed studies (trim and fill method according to Duval and Tweedie).

^b^Fixed model assumed due to non-significant Q-value.

**Table 3.** FU I-III Between-Group Effect Sizes for PTSS, Depression, Anxiety and Grief.

Assessment Point	Outcome	Sample	*n*	g	95% CI	Se	z	Q	I^2^	Duval and Tweedie
FU I	PTSS	Any control	4	(.39^a,b^) .28^a,b,c^	(.04–.73) (-.03 - .60)	(.18)	(2.20*)	2.59	0	1
	Depression	Any control	4	.20^a,b^	−.14–.54	.17	1.14	.62	0	0
FU II	PTSS	TAU/AT	7	.05	−.30–.41	.18	.30	16.70**	64.07	0
	Depression	TAU/AT	6	.17^a^	−.04 −.37	.10	1.63	5.39	7.17	0
	Anxiety	TAU/AT	4	.10^a^	−.14–.33	.12	.81	4.47	32.82	0
FU III	PTSS	TAU/AT	11	.35	.13–.56	.11	3.11**	28.90**	65.40	0
		Efficacy	6	.32^a^	.11–.52	.11	3.01**	3.37	0	0
		Effectiveness	5	.39	.02–.76	.19	2.08*	25.15***	84.10	0
		Individual	7	.29^a^	.10–.47	.09	3.05**	3.79	0	0
		Group	4	.45	.00–.89	.23	1.96*	23.66***	87.32	0
	Depression	TAU/AT	7	.14^a^	−.05–.32	.09	1.47	1.76	0	0
	Anxiety	TAU/AT	5	.18^a^	−.02–.38	.10	1.76	5.11	21.77	0
	Grief	TAU/AT	5	.33	−.02–.67	.18	1.85	16.60**	75.91	0

*Note.* High risk of bias studies are excluded; *n* = number of included comparisons; PTSS = Posttraumatic Stress Symptoms; TAU/AT = Treatment as usual/active treatment control conditions; Effectiveness = Effectiveness RCTs only; Efficacy = Efficacy RCTs only; Individual = RCTs conducted in an individual treatment setting; Group = RCTs conducted in a group setting. *p < .05; **p < .01; ***p < .001.

^a^Fixed model assumed due to non-significant Q-value.

^b^Analysis includes high risk of bias studies (calculation otherwise not possible due to low number of studies).

^c^Recalculated with imputed studies (trim and fill method according to Duval and Tweedie).

**Table 4.** Within-Group and Between-Group Effect Sizes for Caregiver-Reported PTSS, Depression and Anxiety.

Analysis	Assessment Point	Outcome	*n*	g	95% CI	Se	z	Q	I^2^	Duval and Tweedie
Within-group	Pre-post	PTS	18	1.15	.86–1.44	.15	7.68***	177.87***	90.44	0
		Depression	7	(.57) .50^a^	(.33–.82) .23–.76	(.13)	(4.58***)	14.79*	59.44	1
		Anxiety	3	.52^b,c^	.34–.71	.09	5.55***	.08	0	0
	Pre-FU I	PTSS	4	(.89) .71^a^	(.45–1.33) .22–1.21	(.23)	(3.95***)	12.69**	76.35	1
		Depression	3	.57^b^	.36–.78	.11	5.31***	5.10	60.76	0
	Pre-FU II	PTSS	3	.88^c^	.30–.1.46	.09	2.96**	12.86**	84.45	0
	Pre-FU III	PTSS	6	2.02	1.34–2.69	.34	5.86***	77.37***	93.54	0
Between-group	Post	PTSS	7	.59	.19–.98	.20	2.89**	50.62***	88.15	0
		Depression	3	.31^b^	−.02–.64	.17	1.87	3.58	44.14	0

*Note.* High risk of bias studies are excluded; *n* = number of included comparisons; PTSS = Posttraumatic Stress Symptoms.

*p < .05; **p < .01; ***p < .001.

^a^Recalculated with imputed studies (trim and fill method according to Duval and Tweedie).

^b^Fixed model assumed due to non-significant Q-value.

^c^Analysis includes high risk of bias studies (calculation otherwise not possible due to low number of studies).

**Table 5.** Descriptive Statistics, Intraclass Correlations and t-Statistics for Self-Reported and Caregiver-Reported Outcomes.

Outcome	Assessment Points	Assessor	*n*	M	Sd	r	t	p
PTSS	Pre	Participants	28	33.01	8.67	.74	1.10	.28
		Caregivers		30.10	11.02			
	Post	Participants	28	19.65	8.98	.94	.42	.68
		Caregivers		18.55	10.76			
	FU I	Participants	4	13.78	6.56	.95	.07	.95
		Caregivers		13.45	7.84			
	FU III	Participants	6	11.08	7.45	.99	.37	.72
		Caregivers		9.47	7.66			
Depression	Pre	Participants	10	10.46	1.58	.38	1.91	.07
		Caregivers		8.81	2.24			
	Post	Participants	10	6.43	2.62	.91	.90	.38
		Caregivers		5.43	2.36			
	FU I	Participants	3	5.52	4.23	.99	.05	.96
		Caregivers		5.35	3.95			
Anxiety	Pre	Participants	3	23.91	15.72	.89	.55	.61
		Caregivers		17.89	10.68			
	Post	Participants	3	14.63	8.87	.97	.36	.74
		Caregivers		12.23	7.39			

*Note.* n = number of included self-reported and caregiver-reported means; r = intraclass correlation between self-reported and caregiver-reported outcome at the respective assessment point.

### Participant-reported Outcomes

#### PTSS within-Group Effects

Within TF-CBT, uncontrolled effect sizes for PTSS were large from pre to all follow-up assessment points (FU I: g = 1.63, CI 1.22–2.04; FU II: g = 1.65, CI 1.07–2.24; FU III: g = 1.71, CI 1.27–2.15). In comparison to the pre-post effect sizes of the respective studies involved in the analyses, effect sizes were stable and even increased at FU II and FU III. In individual settings at the primary endpoint (FU III), the large effect size (g = 1.63, CI 1.07–2.19) was only slightly smaller than the overall effect size as all but two studies were conducted in individual settings. Accordingly, effect sizes for group settings came from two studies only, showing a large effect (g = 2.47, CI 2.25–2.69) that is considerably greater than the overall effect size and the effect size in individual studies. The results for RCTs were, again, almost identical (g = 1.71, CI 1.24.–2.18) to the overall and individual settings results as only two of the included studies were uncontrolled and all but one RCT were conducted in an individual setting. Effectiveness (g = 2.00, CI 1.53–2.47) and efficacy (g = 1.69, CI .80–2.57) trials both showed large effect sizes with effectiveness studies showing a somewhat greater effect size. In addition, a direct comparison between follow-up assessment points was possible from FU II to FU III showing a small significant increase in the effect size (g = .20, CI .07–.33). For this analysis, all eligible studies were RCTs. The overlap of studies included in FU I and later assessment points was limited to one and two studies, respectively and thus effect sizes could not be calculated.

#### Secondary Outcomes within-Group Effects

Across all follow-up assessment points within TF-CBT, effect sizes were medium to large for depression (FU I: g = .89, CI .54–1.24; FU II: g = .60, CI .48–.73; FU III: g = .99, CI .70–1.29) and small to large for anxiety (FU I: g = .38, CI .17–.59; FU II: g = .82, CI .52–1.11; FU III: g = .95, CI .55–1.35). However, the small effect size for anxiety at FU I relied on three studies only and needed to include studies with a high risk of bias in order to achieve a sufficient number of comparisons for analysis. We were able to calculate grief only at FU III and found a large effect size (g = 1.44, CI .94–1.94). Compared to pre-post effect sizes of the studies involved in the analyses, effect sizes were stable and even increased at FU I (depression), FU II (depression and anxiety) and FU III (depression and anxiety). From FU II to FU III, no significant change was observable for secondary outcomes.

#### PTSS Between-Groups Effects

In comparison to control groups, effect sizes for PTSS were in favor of TF-CBT and small across follow-up assessment points (FU I: g = .28, CI -.03 - .60; FU II: g = .05, CI -.30 - .41; FU III: g = .35, CI .13–.56). However, the difference was not significant at FU II. It should also be noted that at FU I, only two studies contributed effect sizes of which one was a high risk of bias study using a wait list condition as comparator. It was included in the analysis to reach a sufficient number of comparisons. All other analyses used TAU/AT control groups only. Compared to the post-treatment effect size of the respective studies included in the analyses, the effect size decreased at FU I and FU II but was stable at FU III. At FU III, the small effects were paralleled in individual settings (g = .29, CI .10–.47), group settings (g = .45, CI .00–.89), efficacy (g = .32, CI .11–.52) and effectiveness (g = .39, CI .02–.76) trials.

#### Secondary Outcomes Between-Groups Effects

In comparison to control groups across all follow-up assessment points, effect sizes were small and non-significant for depression (FU I-III), anxiety (FU II-III) and grief (FU III). Interestingly, grief was approaching significance (*p* = .06). However, for the studies involved in the follow-up analyses, non-significant differences were already observable at post-treatment in some instances. In terms of effect sizes, follow-up effects were comparable to post-treatment, except for depression showing somewhat smaller effect sizes at FU II and FU III.

### Caregiver-Reported Outcomes

Within TF-CBT, effect sizes for PTSS were large at post-treatment (g = 1.15, CI .86–1.44) and at the primary endpoint (FU III: g = 2.02, CI 1.34–2.69). At FU I (FU I: g = .71, CI .22–1.21) and FU II (g = .88, CI .30–1.46), within-group effect sizes were somewhat smaller but relied on fewer studies. For FU II, analysis had to include high risk of bias studies to achieve a sufficient number of comparisons. Considering the pre-post effect sizes of the respective studies involved in the follow-up analyses, caregiver-reported effects were stable at FU I and FU II and increased at the primary endpoint. For the secondary outcomes, within-group effect sizes were medium across the available assessment points and outcomes (depression post: g = .50, CI .23–.76; FU I: g = .57, CI .36–.78; anxiety post: g = .52, CI .34–.71). The within-group effect size for depression at FU I was stable when compared to the pre-post effect size of studies involved in the analysis. For grief, no caregiver-reports were reported.

For caregiver-reported outcomes, between-group effect sizes could only be computed at post-treatment. In comparison to control conditions, a medium effect size was found for PTSS supporting TF-CBT. For depression, a small effect size was found in favor of TF-CBT. However, it was only approaching significance (*p* = .06).

#### Concordance of Self-Reported and Caregiver-Reported Outcomes

Looking at the agreement between participants and their caregivers, t-tests showed no significant differences between self-reported and caregiver-reported outcomes across assessment points. However, depression was approaching a significant difference at baseline (*p* = .07) and a trend was visible with caregivers consistently producing scores somewhat lower than participants across outcomes. This gap between raters narrowed over time as participants improved. In terms of intraclass correlations, reliability was excellent for most assessment points but tended to be weaker at baseline (PTSS: *r* = .74; depression: *r* = .38; anxiety: *r* = .89) and greater at post-treatment (PTSS: *r* = .94; depression: *r* = .91; anxiety: *r* = .97), FU I (PTSS: *r* = .95; depression: *r* = .99) and FU III (PTSS: *r* = .99). Notably, reliability was poor for depression at baseline. The follow-up analyses and anxiety analyses should be interpreted with caution due to the substantially lower number of included studies. Furthermore, we included high risk of bias studies since we believed risk of bias to be equal for participant and caregiver outcomes and thus not affecting their relationship.

## Discussion

With this meta-analysis, we evaluated the stability of treatment effects of specific TF-CBT from pre to 12-month follow-up and assessed caregiver-reported outcomes as well as their concordance with self-reports. This closes two important gaps in the literature, as it is the only recent meta-analysis for specific TF-CBT on long-term outcomes and the first on caregiver-reports in this context. Additionally, intermediate follow-up assessment points were considered and sub-group analyses were performed for RCTs, individual and group settings as well as effectiveness and efficacy trials at 12-month follow-up. Results showed firm support for specific TF-CBT and indicated high concordance between self-reported and caregiver-reported outcomes. For PTSS, effects were stronger than for secondary outcomes and in comparison to efficacy trials and individual settings, effects were more pronounced in effectiveness trials and group settings. Furthermore, specific TF-CBT outperformed treatment as usual and active treatments with regard to PTSS but not secondary outcomes. Results for RCTs only were highly similar to the overall results.

### PTSS

To the best of our knowledge, no other meta-analysis has investigated uncontrolled effect sizes for specific TF-CBT or similar variants at follow-up. The large uncontrolled effect sizes found from pre to all follow-up assessment points confirmed the stability of treatment effects. Moreover, considering the pre-post effect sizes of the studies involved in the respective analyses, we observed further gains at later assessment points and even from FU II to FU III, supporting the notion that some treatment effects may unfold over time (Tutus et al., 2017).

At the primary endpoint, we found a small controlled effect size favoring TF-CBT compared to TAU/AT conditions. This fits the previous analysis of specific TF-CBT (Cary & McMillen, 2012) but not that of TF-CBT therapies (Morina et al., 2016) which found no significant differences to active treatments. In line with the latter analysis, we also did not observe a significant difference at the FU II intermediate assessment point. However, FU II included fewer comparisons and all but one came from studies using individual treatment settings, which also showed smaller effects at the primary endpoint and in our previous analysis (Thielemann et al., 2022). Thus, the FU II intermediate assessment point was less robust and more homogenous with regard to treatment setting than the primary endpoint. In addition, the findings reported by Morina et al. (2016) are not directly comparable to our results since their analysis combined follow-up periods between 3 and 24 month. In light of the results for the uncontrolled effect sizes, a possible explanation could be that at the earlier follow-up assessment points, TF-CBT may not have fully taken effect yet with TAU/AT conditions temporarily catching up. Unfortunately, the small controlled effect size at FU I could not be compared to earlier findings since none included a mixture of wait list and TAU/AT conditions. We also could not analyze wait list conditions at any follow-up assessment point, since most of them naturally expired. Besides, we are aware of only two studies assessing even longer follow-up periods than our primary endpoint but unfortunately, data was insufficient for meta-analysis (Deblinger et al., 1999; Jensen et al., 2017). In sum, the small effect size at FU III supporting specific TF-CBT over AT/TAU conditions emphasizes its advantage over other treatments as these control groups may themselves be very effective (Frost et al., 2014).

In contrast to the assumption that results from efficacy trials are difficult to transfer into practice (Singal et al., 2014), we found a somewhat greater effect size for effectiveness trials. Additionally, contrary to earlier results (Gutermann et al., 2016), we observed a greater effect size in group settings than individual settings. We discussed this phenomenon in detail in our previous meta-analysis (see Thielemann et al., 2022). In short, specific TF-CBT efficacy trials have so far mostly included studies conducted in an individual setting while effectiveness trials more often included studies conducted in group settings. Group settings showed stronger effects that may explain the counterintuitive difference in favor of effectiveness trials. The stronger effects for group settings can be partly explained by higher baseline symptomatology in underserved populations. However, this cannot fully account for these greater effects and we hypothesized that a specific group factor might be at play that may favor trauma-focused work and consequent recovery in young people. That this pattern of results is also observable at FU III underlines that specific TF-CBT in group settings is a cost-effective timesaving option, especially if resources are limited (Dorsey et al., 2020). It may be used to address mass casualty events affecting many children and adolescents such as pandemics, wars, natural disasters and terrorist attacks.

### Secondary Outcomes

Again, this meta-analysis is unique in investigating uncontrolled effect sizes for specific TF-CBT at follow-up for secondary outcomes of depression, anxiety and grief. Stable treatment effects were confirmed by the small to large uncontrolled effect sizes found from pre to all follow-up assessment points. Regarding the effect sizes from pre to the primary endpoint only, effect sizes were large for all secondary outcomes. Considering the pre-post effect sizes of the studies involved in the respective analyses, further gains were observed at later assessment except from FU II to FU III. This finding further supports the argument that participants continue to improve after treatment, also for secondary outcomes (Cohen et al., 2005; Tutus et al., 2017).

Concerning controlled effect sizes of secondary outcomes, only depression was investigated by previous meta-analyses. Our finding of a small non-significant controlled effect sizes compared to TAU/AT conditions at the primary endpoint is in line with the results of Cary and McMillen (2012) who also found a small non-significant effect for depression at 12-month follow-up compared to active non-CBT conditions. In contrast, Morina et al. (2016) found a medium effect size at 3–24-month follow-up in favor of TF-CBT in comparison to active control conditions (i.e. not active treatments). As stated above, it is difficult to draw a direct comparison between studies due to the differing follow-up periods and the other manuals that were included in their analysis. In addition, the greater effect size can be explained because active treatments were not part of the control condition.

Nevertheless, the disappearance of all significant controlled effect sizes for all secondary outcomes at all follow-up assessment points in our analyses was rather surprising considering the strong pre to FU III effects as well as the post-treatment effects in favor of specific TF-CBT in our earlier analysis (Thielemann et al., 2022). One possible explanation is that the study sample was different in the present analysis. While we drew them from the same pool as our previous analysis, fewer studies included follow-up assessment points and control groups, making it more difficult to detect significant effects. Supporting this hypothesis, the studies with follow-up asssessments, while reporting effect sizes comparable to our previous analyses, typically had fewer significant effects. However, another explanation could be that other treatments are simply equally effective in the long run with regard to secondary symptoms. In addition, TF-CBT mainly targets PTSS and thus smaller effects can be expected for secondary outcomes. Interestingly, grief was approaching significance at the primary endpoint warranting further investigation as the available evidence came from two studies only and no reliable pediatric grief instrument was available.

### Caregiver-Reported Outcomes

In terms of effect sizes, caregiver-reports paralleled the effects found in the pre-post and pre-FU III analyses of participants for all outcomes and were also concordant with participant outcomes in our previous analysis (Thielemann et al., 2022). The controlled post-treatment effect sizes against any control groups were also comparable to our previous results. In line with that, we found no significant differences between raters and their reliability was high across outcomes and assessment points. This finding is contrary to earlier meta-analyses that only found low to moderate agreements between caregivers and their children and adolescents (Achenbach et al., 1987; Los Reyes et al., 2015). Nevertheless, some individual studies in children and adolescents with physical illnesses found similar results (Clawson et al., 2013; Erickson et al., 2017; Phipps et al., 2005; Stevanovic et al., 2012). One explanation could be that caregivers are more aware of their children’s and adolescents’ mental health concerns and actively seek treatment for them. However, it could also be that caregivers strongly attribute problems to their children and adolescents or even report greater symptoms to emphasize treatment needs (Asbrand et al., 2021). Some literature also suggests that greater parental distress and trauma symptoms also result in more symptoms being reported for their children (Exenberger et al., 2019; Schreier et al., 2005; Shemesh et al., 2005). However, since we found that reports converged over time with the treatment being completed and symptoms decreasing, this suggests that the greater concordance between raters in specific TF-CBT may be facilitated by caregivers being directly engaged in treatment. Thereby, they may gain awareness for their youth’s symptoms and possibly learn techniques that also help them to deal with their own symptomatology. Furthermore, as a consequence of treatment participation, caregivers’ greater understanding of PTSS may help them accommodate their children’s behaviors given their condition.

In line with that, there was one exception to high rater agreement with depression showing poor reliability at baseline and excellent reliability at later assessment points. At baseline, the difference between raters was close to significance, suggesting that caregivers might not be suitable to rate their children’s and adolescents’ depression before treatment. In other words, caregivers were not fully aware of the extent of depressive symptoms the young people experienced prior to treatment initiation. This is in line with the literature suggesting that caregivers have more difficulties to assess internalizing symptoms as they are often more difficult to observe (Achenbach et al., 1987; Los Reyes et al., 2015).

Although concordance between self-reports and caregiver-reports was high, we based our analysis on total scores. Thus, on a diagnostic level, we did not evaluate concordance with regard to symptom clusters and diagnoses as these information were mostly unavailable. Earlier studies found that diagnostic agreement was often limited (Choudhury et al., 2003; Grills & Ollendick, 2003; Kassam-Adams et al., 2006; Meiser-Stedman et al., 2007) also in studies with moderate to high total score concordance (Clawson et al., 2013; Erickson et al., 2017). Thus, while our findings suggest that either informant is sufficient to assess symptom severity, when arriving at a diagnosis and treatment decisions are made accordingly, it seems advisable to consider both perspectives as they may offer unique information that can assist diagnosis and treatment decisions.

### Limitations

First of all, the presence of publication bias indicated that some smaller effect studies did not get published. We did address this problem in our analyses but unfortunately, the true value of unpublished (grey) literature remains unknown. Furthermore, we might have missed studies that were not recorded by the search terms. Besides that, some studies and assessment points were ultimately excluded, as data could not be obtained. Subsequently, some intermediate analyses were limited to very few studies, individual settings and TAU/AT control conditions. Moreover, follow-up assessments were limited to 12-month post-treatment as data for later assessment points was insufficient. Furthermore, we could assess concordance between caregiver and participant ratings only in terms of total scores but not diagnoses. Another shortcoming was the inclusion of some older studies that sometimes used categorical instruments instead of dimensional ones. In addition, we used many different instruments for the same outcome and thus no minimal symptom criterion was defined. Consequently, baseline assessments varied strongly across studies. Moreover, a reliable instrument for pediatric grief is still lacking.

### Conclusion and Clinical Implications

This meta-analysis confirmed specific TF-CBT as an effective treatment for pediatric PTSS and secondary outcomes of depression, anxiety and grief at 12-month follow-up. In terms of PTSS, specific TF-CBT showed advantages over TAU/AT control conditions at this primary endpoint. However, regarding secondary outcomes, advantages over other treatments disappeared at follow-up. Effectiveness trials also showed favorable results for specific TF-CBT at the 12-month follow-up, confirming that an easy translation into practice is possible with group settings as a feasible timesaving and cost-effective alternative. This provides further support for TF-CBT’s large-scale use and endorsement by international guidelines (Forbes et al., 2020; National Institute for Health and Care Excellence, 2018; Phoenix Australia Centre for Posttraumatic Mental Health, 2013). Besides, caregiver-reports mirrored the findings of our previous analysis (Thielemann et al., 2022) and showed high concordance with self-reported outcomes. No significant difference between raters was detected for any outcome.

In sum, specific TF-CBT should be the first choice of treatment for pediatric PTSS. Group settings may be used to address high treatment demands with limited resources. In the context of specific TF-CBT, self-reports or caregiver-reports can serve to assess symptom severity in children and adolescents when either informant is unavailable. However, caregiver assessments of internalizing symptoms should be treated with caution prior to treatment initiation. Additionally, both perspectives should be considered when diagnoses are derived and treatment arrangements are made as they may each provide unique information that can assist decision-making.

Future TF-CBT studies should more frequently consider follow-up assessments and longer follow-up periods to fully understand its long-term effects and to investigate whether further treatment gains occur. In addition, effects in group settings warrant further investigation also in western countries and populations with good health care available and should be used to provide treatments to large numbers of individuals in a timely manner. If screening instruments assess self-report as well as caregiver-report and derive presumptive diagnoses, they should report them for both raters to further evaluate the diagnostic concordance. Furthermore, a reliable instrument for pediatric grief symptoms needs to be developed and the categorical instrument of older studies should be contrasted with newer dimensional ones.

## Supplemental Material

Supplemental Material - Stability of Treatment Effects and Caregiver-Reported Outcomes: A Meta-Analysis of Trauma-Focused Cognitive Behavioral Therapy for Children and Adolescents Supplemental Material for Stability of Treatment Effects and Caregiver-Reported Outcomes: A Meta-Analysis of Trauma-Focused Cognitive Behavioral Therapy for Children and Adolescents by Barbara Kasparik, Barbara Kasparik, Julia König, Johanna Unterhitzenberger, Rita Rosner in Child Maltreatment
