# Supplementary material for: Stability of Treatment Effects and Caregiver-Reported Outcomes: A Meta-Analysis of Trauma-Focused Cognitive Behavioral Therapy for Children and Adolescents
Source: Child Maltreat. 2023 Mar 23;29(2):375–87. doi: 10.1177/10775595231167383 (PMC10981190; doi:10.1177/10775595231167383)
Supplement: Supplemental Material - Stability of Treatment Effects and Caregiver-Reported Outcomes: A Meta-Analysis of Trauma-Focused Cognitive Behavioral Therapy for Children and Adolescents [file sj-pdf-1-cmx-10.1177_10775595231167383.pdf]

Table 6.

Overview of all Included RCTs.

Author (year)	TF-CBT Conditions (n)	Control Conditions (n)	Design	Participant-reported Outcomes (Instruments)	Proxy-reported Outcomes (Instruments)
Allen et al. (2021)	TF-CBT (n = 16)<br>TF-CBT, animal-assisted (n = 17)	-<br>-	Efficacy	PTSS (UCLA)<br>Depression (SMFQ)<br>Anxiety (SCARED)	PTSS (UCLA)
Brown et al. (2020)	TF-CBT (n = 7)	TAU/AT, Client-Centered Therapy (n = 10)	Efficacy	PTSS (CPSS)<br>Depression (BASC Dep)<br>Anxiety (BASC Anx)<br>Grief (EGI-TG)	
Cohen et al. (2004); Deblinger et al. (2006)	TF-CBT (n = 89)	TAU/AT, Client-Centered Therapy (n = 91)	Efficacy	PTSS (K-SADS[1])<br>Depression (CDI)<br>Anxiety (STAIC-T)	
Cohen & Mannarino (1998); Cohen et al. (2005)	TF-CBT (n = 41)	TAU/AT, Non-directive Supportive Therapy (n = 41)	Efficacy	PTSS (TSCC-PTS)<br>Depression (CDI)<br>Anxiety (STAIC-T)	
Davidson et al. (2019)	TF-CBT (n = 7)<br>TF-CBT, tablet-facilitated (n = 17)	-<br>-	Effectiveness	PTSS (UCLA)<br>Depression (CESD-C)	PTSS (UCLA)
Deblinger et al. (1996); Deblinger et al. (1999)	TF-CBT, child only (n = 21)<br>TF-CBT, child and mother (n = 19)	TAU/AT (n = 20)[2]<br>TAU/AT (n = 20)[2]	Efficacy	PTSS (K-SADS)<br>Depression (CDI)<br>Anxiety (STAIC-T)	
Diehle et al. (2015)	TF-CBT (n = 23)	TAU/AT, Eye Movement Desensitization and Reprocessing (n = 25)	Efficacy	PTSS (CAPS-CA)<br>Depression (RCADS-mdd)<br>Anxiety (RCADS[3])	Depression (RCADS-mdd)<br>Anxiety (RCADS[3])

Dorsey et al. (2020)	TF-CBT Group, Urban Kenya (n = 96)	TAU/AT (n = 96)	Effectiveness	PTSS (CPSS)<br>Grief (ICG)	PTSS (CPSS)
	TF-CBT Group, Rural Kenya (n = 64)	TAU/AT (n = 64)			
	TF-CBT Group, Urban Tanzania (n = 96)	TAU/AT (n = 96)			
	TF-CBT Group, Rural Tanzania (n = 64)	TAU/AT (n = 64)			
Dorsey et al. (2014)	TF-CBT, outcome data merged for both TF-CBT conditions (n = 27)	-	Efficacy	PTSS (UCLA)<br>Depression (CDI)	PTSS (UCLA)
Goldbeck et al. (2016); Tutus et al. (2017)	TF-CBT (n = 76)	WL (n = 83)	Effectiveness	PTSS (CAPS-CA)<br>Depression (CDI)<br>Anxiety (SCARED)	PTSS (UCLA)<br>Anxiety (SCARED)
Jaberghaderi et al. (2004)	TF-CBT (n = 7)	TAU/AT, Eye Movement Desensitization and Reprocessing (n = 7)	Efficacy	PTSS (CROPS)	PTSS (PROPS)
Jensen et al. (2014); Jensen et al. (2017)	TF-CBT (n = 59)	TAU/AT (n = 63)	Effectiveness	PTSS (CPSS[4])<br>Depression (MFQ)<br>Anxiety (SCARED)	
King et al. (2000)	TF-CBT, child only (n = 12)	WL (n = 12)	Efficacy	PTSS (ADIS)<br>Depression (CDI)<br>Anxiety (R-CMAS)	
	TF-CB, child and mother (n = 12)	WL (n = 12)			
Langley et al. (2015)	TF-CBT Group, Bounce Back, immediate treatment (n = 35)	WL (n = 36)	Effectiveness	PTSS (UCLA)<br>Depression (CDI)<br>Anxiety (SCARED)	PTSS (UCLA)<br>Depression (CDI)

	TF-CBT Group, Bounce Back, delayed treatment (n = 34)	-			
McMullen et al. (2013)	TF-CBT Group (n = 24)	WL (n = 24)	Effectiveness	PTSS (UCLA)	
Nixon et al. (2012); Nixon et al. (2017)	TF-CBT (n = 17)	TAU/AT, TF-CBT without exposure (n = 16)[5]	Efficacy	PTSS (CAPS-CA) Depression (CDI) Anxiety (R-CMAS)	
O'Callaghan et al. (2015)	TF-CBT Group (n = 26)	TAU/AT, Child Friendly Spaces (n = 24)	Effectiveness	PTSS (UCLA)	
O'Callaghan et al. (2013)	TF-CBT Group (n = 24)	WL (n = 28)	Effectiveness	PTSS (UCLA)	
Salloum et al. (2015)	TF-CBT (n = 11)	- [6]	Efficacy	PTSS (UCLA)	
Santiago et al. (2018)	TF-CBT Group, Bounce Back, immediate treatment (n = 25)	WL (n = 27)	Effectiveness	PTSS (UCLA) Depression (CDI) Anxiety (SCARED)	PTSS (UCLA) Depression (CDI)
	TF-CBT Group, Bounce Back, delayed treatment (n = 27)	-			
Schottelkorb et al. (2012)	TF-CBT (n = 12)	TAU/AT, Child-Centered Play Therapy (n = 14)	Effectiveness	PTSS (UCLA)	PTSS (PROPS)

Note. Sample size displayed for the respective PTSS measure. TF-CBT = Trauma-focused Cognitive Behavioral Therapy; TAU = Treatment as Usual; AT = Active treatment condition; WL = Wait-list condition; PTSS = Post-traumatic Stress Symptoms; UCLA = UCLA Posttraumatic Stress Disorder-Reaction Index; (S)MFQ = Mood and Feelings Questionnaire, (Short Version); SCARED = Screen for Child Anxiety Related Emotional Disorders; CPSS = Child PTSD Symptom Scale; BASC Dep = Behavioral Assessment System for Children, Depression subscale; Anx = Anxiety subscale; EGI-TG = Extended Grief Inventory-Traumatic Grief Subscale; K-SADS = K-SADS = Schedule for Affective Disorders and Schizophrenia

for School-Age Children; CDI = Children's Depression Inventory; STAIC-T = State-Trait Anxiety Inventory for Children, Trait subscale; TSCC-PTS = Trauma Symptom Checklist for Children, Post-traumatic Stress subscale; CESD-C = Center for Epidemiological Studies Depression Scale for Children; CAPS-CA = Clinician-Administered PTSD Scale for Children and Adolescents; RCADS-mdd = Revised Child Anxiety and Depression Scale, major depressive disorder subscale; ICG = Inventory of Complicated Grief; CROPS = Child Report of Post-traumatic Symptoms; PROPS = Parent Report of Post-traumatic Symptoms; ADIS = Anxiety Disorders Interview Schedule, Child version; R-CMAS = Revised Children's Manifest Anxiety Scale.

[1] Weighted mean and pooled standard deviation for subscales Reexperiencing, Avoidance, Hypervigilance.

[2] Community condition not included.

[3] Weighted mean and pooled standard deviation for subscales Separation Anxiety Disorder, Social Phobia, Panic Disorder, Generalized Anxiety Disorder.

[4] CPSS was preferred over CAPS-CA to allow for follow-up.

[5] TF-CBT without exposure component was regarded as active non-TF-CBT condition.

[6] SC-TF-CBT condition was excluded from analyses as some participants received TF-CBT while others did not.

Table 7.

Overview of all Included Uncontrolled Studies

Author (year)	TF-CBT conditions (n)	Participant-reported Outcomes (Instruments)	Proxy-reported Outcomes (Instruments)
Amaya-Jackson et al. (2018)	TF-CBT (n = 156)	PTSS (UCLA)<br>Depression (CDI)	PTSS (UCLA)
Cohen et al. (2006)	TF-CBT (n = 39)	PTSS (CPSS)<br>Depression (MFQ)<br>Grief (EGI-TG)	PTSS (CPSS)
Cohen et al. (2004)	TF-CBT (n = 22)	PTSS (CPSS)<br>Depression (MFQ)<br>Anxiety (SCARED)<br>Grief (EGI-TG)	PTSS (CPSS)
Espeleta et al. (2021)	TF-CBT (n = 99)	PTSS (CPSS)<br>Depression (SMFQ)	PTSS (CPSS)
Kirsch et al. (2011)	TF-CBT (n = 13)	PTSS (CAPS-CA)<br>Depression (CDI)<br>Anxiety (SCARED)	PTSS (UCLA)
Konanur et al. (2015)	TF-CBT (n = 56)	PTSS (TSCC-PTS[1])	PTSS (TSCYC)
Lang et al. (2015)	TF-CBT (n = 188)	PTSS (UCLA)<br>Depression (SMFQ)	
O'Donnel et al. (2014)	TF-CBT Group (n = 64)	PTSS (UCLA)<br>Depression (SMFQ)<br>Grief (GSS)	PTSS (UCLA)<br>Depression (SMFQ)
	TF-CBT, DSM-V (n =26)	PTSS (CPSS)	
Stewart et al. (2020)	TF-CBT, DSM-IV (n = 41)	PTSS (UCLA)<br>Depression (SMFQ)	PTSS (UCLA)<br>Depression (SMFQ)

	TF-CBT, DSM-V (n = 18)	PTSS (UCLA)<br>Depression (SMFQ)	PTSS (UCLA)<br>Depression (SMFQ)
Stewart et al. (2017)	TF-CBT (n = 15)	PTSS (UCLA)<br>Depression (SMFQ)<br>Anxiety (SCARED)	PTSS (UCLA)<br>Depression (SMFQ)<br>Anxiety (SCARED)
Unterhitzenberger et al. (2019)	TF-CBT (n = 26)	PTSS (CATS)<br>Depression (MFQ)	PTSS (CATS)<br>Depression (MFQ)
Webb et al. (2014)	TF-CBT (n = 70)	PTSS (UCLA)	
	TF-CBT Group USA (n = 4)	PTSS (UCLA)<br>Depression (CDI)	

Note. Sample size displayed for the respective PTSS measure. TF-CBT = Trauma-focused Cognitive Behavioral Therapy; PTSS = Post-traumatic Stress Symptoms; UCLA = UCLA Posttraumatic Stress Disorder-Reaction Index; CDI = Children's Depression Inventory; CPSS = Child PTSD Symptom Scale; (S)MFQ = Mood and Feelings Questionnaire, (Short Version); EGI-TG = Extended Grief Inventory-Traumatic Grief Subscale; SCARED = Screen for Child Anxiety Related Emotional Disorders; TSCC-PTS = Trauma Symptom Checklist for Children, Post-traumatic Stress subscale; TSCYC-PTS = Trauma Symptom Checklist for Young Children, Post-traumatic Stress subscale; CATS = Child and Adolescent Trauma Screen.

[1] Standard Deviation for baseline (pre-therapy) retrieved from earlier assessment point (pre-assessment).

References

Allen, B., Shenk, C. E., Dreschel, N. E., Wang, M., Bucher, A. M., Desir, M. P., . . . Grabowski, S. R. (2021). Integrating Animal-Assisted Therapy Into TF-CBT for Abused Youth With PTSD: A Randomized Controlled Feasibility Trial. *Child Maltreatment.* Advance online publication. https://doi.org/10.1177/1077559520988790

Amaya-Jackson, L., Hagele, D., Sideris, J., Potter, D., Briggs, E. C., Keen, L., . . . Socolar, R. (2018). Pilot to policy: Statewide dissemination and implementation of evidence-based treatment for traumatized youth. *BMC Health Services Research*, *18*(1), 589. https://doi.org/10.1186/s12913-018-3395-0

Brown, E. J., Goodman, R. F., Cohen, J. A., Mannarino, A. P., & Chaplin, W. F. (2020). An Exploratory Trial of Cognitive-Behavioral vs Client-Centered Therapies for Child-Mother Dyads Bereaved from Terrorism. *Journal of Child & Adolescent Trauma*, *13*(1), 113–125. https://doi.org/10.1007/s40653-019-00264-2

Cohen, J. A., Deblinger, E., Mannarino, A. P., & Steer, R. A. (2004). A multisite, randomized controlled trial for children with sexual abuse-related PTSD symptoms. *Journal of the American Academy of Child and Adolescent Psychiatry*, *43*(4), 393–402. https://doi.org/10.1097/00004583-200404000-00005

Cohen, J. A., & Mannarino, A. P. (1998). Interventions for Sexually abused Children: Initial Treatment Outcome Findings. *Child Maltreatment*, *3*(1), 17–26. https://doi.org/10.1177/1077559598003001002

Cohen, J. A., Mannarino, A. P., & Knudsen, K. (2004). Treating childhood traumatic grief: A pilot study. *Journal of the American Academy of Child and Adolescent Psychiatry*, *43*(10), 1225–1233. https://doi.org/10.1097/01.chi.0000135620.15522.38

Cohen, J. A., Mannarino, A. P., & Knudsen, K. (2005). Treating sexually abused children: 1 year follow-up of a randomized controlled trial. *Child Abuse & Neglect*, *29*(2), 135–145. https://doi.org/10.1016/j.chiabu.2004.12.005

Cohen, J. A., Mannarino, A. P., & Staron, V. R. (2006). A pilot study of modified cognitive-behavioral therapy for childhood traumatic grief (CBT-CTG). *Journal of the American Academy of Child and Adolescent Psychiatry*, *45*(12), 1465–1473. https://doi.org/10.1097/01.chi.0000237705.43260.2c

Davidson, T. M., Bunnell, B. E., Saunders, B. E., Hanson, R. F., Danielson, C. K., Cook, D., . . . Ruggiero, K. J. (2019). Pilot Evaluation of a Tablet-Based Application to Improve Quality of Care in Child Mental Health Treatment. *Behavior Therapy*, *50*(2), 367–379. https://doi.org/10.1016/j.beth.2018.07.005

Deblinger, E., Lippmann, J., & Steer, R. (1996). Sexually Abused Children Suffering Posttraumatic Stress Symptoms: Initial Treatment Outcome Findings. *Child Maltreatment*, *1*(4), 310–321. https://doi.org/10.1177/1077559596001004003

Deblinger, E., Mannarino, A. P., Cohen, J. A., & Steer, R. A. (2006). A follow-up study of a multisite, randomized, controlled trial for children with sexual abuse-related PTSD symptoms. *Journal of the American Academy of Child and Adolescent Psychiatry*, *45*(12), 1474–1484. https://doi.org/10.1097/01.chi.0000240839.56114.bb

Deblinger, E., Steer, R. A., & Lippmann, J. (1999). Two-year follow-up study of cognitive behavioral therapy for sexually abused children suffering post-traumatic stress symptoms. *Child Abuse & Neglect*, *23*(12), 1371–1378. https://doi.org/10.1016/S0145-2134(99)00091-5

Diehle, J., Opmeer, B. C., Boer, F., Mannarino, A. P., & Lindauer, R. J. L. (2015). Trauma-focused cognitive behavioral therapy or eye movement desensitization and reprocessing: What works in children with posttraumatic stress symptoms? A randomized controlled trial. *European Child & Adolescent Psychiatry*, *24*(2), 227–236. https://doi.org/10.1007/s00787-014-0572-5

Dorsey, S., Lucid, L., Martin, P., King, K. M., O'Donnell, K., Murray, L. K., . . . Whetten, K. (2020). Effectiveness of Task-Shifted Trauma-Focused Cognitive Behavioral Therapy for Children Who Experienced Parental Death and Posttraumatic Stress in Kenya and Tanzania: A Randomized Clinical Trial. *JAMA Psychiatry*, *77*(5), 464–473. https://doi.org/10.1001/jamapsychiatry.2019.4475

Dorsey, S., Pullmann, M. D., Berliner, L., Koschmann, E., McKay, M., & Deblinger, E. (2014). Engaging foster parents in treatment: A randomized trial of supplementing trauma-focused cognitive behavioral therapy with evidence-based engagement strategies. *Child Abuse & Neglect*, *38*(9), 1508–1520. https://doi.org/10.1016/j.chiabu.2014.03.020

Espeleta, H. C., Peer, S. O., Are, F., & Hanson, R. F. (2021). Therapists' Perceived Competence in Trauma-Focused Cognitive Behavioral Therapy and Client Outcomes: Findings From a Community-Based Learning Collaborative. *Child Maltreatment*, 10775595211003673. https://doi.org/10.1177/10775595211003673

Goldbeck, L., Muche, R., Sachser, C., Tutus, D., & Rosner, R. (2016). Effectiveness of Trauma-Focused Cognitive Behavioral Therapy for Children and Adolescents: A Randomized Controlled Trial in Eight German Mental Health Clinics. *Psychotherapy and Psychosomatics*, *85*(3), 159–170. https://doi.org/10.1159/000442824

Jaberghaderi, N., Greenwald, R., Rubin, A., Zand, S. O., & Dolatabadi, S. (2004). A comparison of CBT and EMDR for sexually-abused Iranian girls. *Clinical Psychology & Psychotherapy*, *11*(5), 358–368. https://doi.org/10.1002/cpp.395

Jensen, T. K., Holt, T., & Ormhaug, S. M. (2017). A Follow-Up Study from a Multisite, Randomized Controlled Trial for Traumatized Children Receiving TF-CBT. *Journal of Abnormal Child Psychology*, *45*(8), 1587–1597. https://doi.org/10.1007/s10802-017-0270-0

Jensen, T. K., Holt, T., Ormhaug, S. M., Egeland, K., Granly, L., Hoaas, L. C., . . . Wentzel-Larsen, T. (2014). A randomized effectiveness study comparing trauma-focused cognitive behavioral therapy with therapy as usual for youth. *Journal of Clinical Child and Adolescent Psychology : The Official Journal for the Society of Clinical Child and Adolescent Psychology, American Psychological Association, Division 53*, *43*(3), 356–369. https://doi.org/10.1080/15374416.2013.822307

King, N. J., Tonge, B. J., Mullen, P., Myerson, N., Heyne, D., Rollings, S., . . . Ollendick, T. H. (2000). Treating sexually abused children with posttraumatic stress symptoms: A randomized clinical trial. *Journal of the American Academy of Child and Adolescent Psychiatry*, *39*(11), 1347–1355. https://doi.org/10.1097/00004583-200011000-00008

Kirsch, V., Fegert, J. M., Seitz, D. C. M., & Goldbeck, L. (2011). Traumafokussierte kognitive Verhaltenstherapie (Tf-KVT) bei Kindern und Jugendlichen nach Missbrauch und Misshandlung. *Kindheit Und Entwicklung*, *20*(2), 95–102. https://doi.org/10.1026/0942-5403/a000045

Konanur, S., Muller, R. T., Cinamon, J. S., Thornback, K., & Zorzella, K. P. M. (2015). Effectiveness of Trauma-Focused Cognitive Behavioral Therapy in a community-based program. *Child Abuse & Neglect*, *50*, 159–170. https://doi.org/10.1016/j.chiabu.2015.07.013

Lang, J. M., Franks, R. P., Epstein, C., Stover, C., & Oliver, J. A. (2015). Statewide dissemination of an evidence-based practice using Breakthrough Series Collaboratives. *Children and Youth Services Review*, *55*, 201–209. https://doi.org/10.1016/j.childyouth.2015.06.005

Langley, A. K., Gonzalez, A., Sugar, C. A., Solis, D., & Jaycox, L. (2015). Bounce back: Effectiveness of an elementary school-based intervention for multicultural children exposed to traumatic events. *Journal of Consulting and Clinical Psychology*, *83*(5), 853–865. https://doi.org/10.1037/ccp0000051

McMullen, J., O'Callaghan, P., Shannon, C., Black, A., & Eakin, J. (2013). Group trauma-focused cognitive-behavioural therapy with former child soldiers and other war-affected boys in the DR Congo: A randomised controlled trial. *Journal of Child Psychology and Psychiatry, and Allied Disciplines*, *54*(11), 1231–1241. https://doi.org/10.1111/jcpp.12094

Nixon, R., Sterk, J., & Pearce, A. (2012). A randomized trial of cognitive behaviour therapy and cognitive therapy for children with posttraumatic stress disorder following single-incident trauma. *Journal of Abnormal Child Psychology*, *40*(3), 327–337. https://doi.org/10.1007/s10802-011-9566-7

Nixon, R., Sterk, J., Pearce, A., & Weber, N. (2017). A randomized trial of cognitive behavior therapy and cognitive therapy for children with posttraumatic stress disorder following single-incident trauma: Predictors and outcome at 1-year follow-up. *Psychological Trauma : Theory, Research, Practice and Policy*, *9*(4), 471–478. https://doi.org/10.1037/tra0000190

O'Callaghan, P., McMullen, J., Shannon, C., & Rafferty, H. (2015). Comparing a trauma focused and non trauma focused intervention with war affected Congolese youth. *Intervention*, *13*(1), 28–44. https://doi.org/10.1097/WTF.0000000000000054

O'Callaghan, P., McMullen, J., Shannon, C., Rafferty, H., & Black, A. (2013). A randomized controlled trial of trauma-focused cognitive behavioral therapy for sexually exploited, war-affected Congolese girls. *Journal of the American Academy of Child and Adolescent Psychiatry*, *52*(4), 359–369. https://doi.org/10.1016/j.jaac.2013.01.013

O'Donnell, K., Dorsey, S., Gong, W., Ostermann, J., Whetten, R., Cohen, J. A., . . . Whetten, K. (2014). Treating maladaptive grief and posttraumatic stress symptoms in orphaned children in Tanzania: Group-based trauma-focused cognitive-behavioral therapy. *Journal of Traumatic Stress*, *27*(6), 664–671. https://doi.org/10.1002/jts.21970

Salloum, A., Small, B. J., Robst, J., Scheeringa, M. S., Cohen, J. A., & Storch, E. A. (2015). Stepped and Standard Care for Childhood Trauma. *Research on Social Work Practice*, *27*(6), 653–663. https://doi.org/10.1177/1049731515601898

Santiago, C. D., Raviv, T., Ros, A. M., Brewer, S. K., Distel, L. M. L., Torres, S. A., . . . Langley, A. K. (2018). Implementing the Bounce Back trauma intervention in urban elementary schools: A real-world replication trial. *School Psychology Quarterly : The Official Journal of the Division of School Psychology, American Psychological Association*, *33*(1), 1–9. https://doi.org/10.1037/spq0000229

Schottelkorb, A. A., Doumas, D. M., & Garcia, R. (2012). Treatment for childhood refugee trauma: A randomized, controlled trial. *International Journal of Play Therapy*, *21*(2), 57–73. https://doi.org/10.1037/a0027430

Stewart, R. W., Orengo-Aguayo, R. E., Cohen, J. A., Mannarino, A. P., & Arellano, M. A. de (2017). A Pilot Study of Trauma-Focused Cognitive-Behavioral Therapy Delivered via Telehealth Technology. *Child Maltreatment*, *22*(4), 324–333. https://doi.org/10.1177/1077559517725403

Stewart, R. W., Orengo-Aguayo, R., Young, J., Wallace, M. M., Cohen, J. A., Mannarino, A. P., & Arellano, M. A. de (2020). Feasibility and effectiveness of a telehealth service delivery model for treating childhood posttraumatic stress: A community-based, open pilot trial of trauma-focused cognitive–behavioral therapy. *Journal of Psychotherapy Integration*, *30*(2), 274–289. https://doi.org/10.1037/int0000225

Tutus, D., Pfeiffer, E., Rosner, R., Sachser, C., & Goldbeck, L. (2017). Sustainability of Treatment Effects of Trauma-Focused Cognitive-Behavioral Therapy for Children and Adolescents: Findings from 6- and 12-Month Follow-Ups. *Psychotherapy and Psychosomatics*, *86*(6), 379–381. https://doi.org/10.1159/000481198

Unterhitzenberger, J., Wintersohl, S., Lang, M., König, J., & Rosner, R. (2019). Providing manualized individual trauma-focused CBT to unaccompanied refugee minors with uncertain residence status: A pilot study. *Child and Adolescent Psychiatry and Mental Health*, *13*, 22. https://doi.org/10.1186/s13034-019-0282-3

Webb, C., Hayes, A., Grasso, D., Laurenceau, J.-P., & Deblinger, E. (2014). Trauma-Focused Cognitive Behavioral Therapy for Youth: Effectiveness in a Community Setting. *Psychological Trauma : Theory, Research, Practice and Policy*, *6*(5), 555–562. https://doi.org/10.1037/a0037364

Table 8.

PTSS Pre-FU III Within-Group Effect Size.

Study	Condition	Outcome	Hedges's g	Standard error	Variance	Lower limit	Upper limit	Z-Value	p-Value
Brown et al. 2020	TF-CBT	PTSS	0,077	0,363	0,132	-0,635	0,788	0,211	0,833
Cohen et al. 1998; 2005	TF-CBT	PTSS	0,717	0,189	0,036	0,347	1,088	3,797	0,000
Cohen, Deblinger et al. 2004; Deblinger et al. 2006	TF-CBT	PTSS	2,374	0,239	0,057	1,906	2,842	9,945	0,000
Deblinger et al., 1996; 1999	TF-CBT (child only)	PTSS	1,566	0,351	0,123	0,879	2,253	4,466	0,000
Deblinger et al., 1996; 1999	TF-CBT (mother and child)	PTSS	2,299	0,474	0,225	1,369	3,228	4,848	0,000
Dorsey et al. 2020	TF-CBT group (KR)	PTSS	2,173	0,250	0,063	1,683	2,663	8,687	0,000
Dorsey et al. 2020	TF-CBT group (KU)	PTSS	2,215	0,207	0,043	1,808	2,621	10,685	0,000
Dorsey et al. 2020	TF-CBT group (TR)	PTSS	2,594	0,285	0,081	2,035	3,153	9,093	0,000
Dorsey et al. 2020	TF-CBT group (TU)	PTSS	2,942	0,258	0,066	2,437	3,447	11,417	0,000
Goldbeck et al. 2016; Tutus et al. 2017	TF-CBT	PTSS	1,455	0,165	0,027	1,132	1,778	8,831	0,000
Jensen et al. 2014, 2017	TF-CBT	PTSS	1,661	0,253	0,064	1,165	2,157	6,569	0,000
Nixon et al. 2012; 2017	TF-CBT	PTSS	3,320	0,510	0,261	2,319	4,320	6,503	0,000
O'Donnel et al. 2014	TF-CBT group	PTSS	2,658	0,298	0,089	2,075	3,242	8,926	0,000
Webb et al., 2014	TF-CBT	PTSS	1,342	0,185	0,034	0,980	1,704	7,263	0,000
			1,931	0,211	0,044	1,518	2,345	9,154	0,000

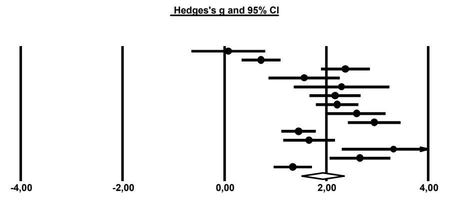

KR = Kenia Rural; KU = Kenia Urban; TR = Tanzania Rural; TU = Tanzania Urban.

Table 9.

Depression Pre-FU III Within-Group Effect Size.

Study name	Condition	Outcome	Hedges's g	Standard error	Variance	Lower limit	Upper limit	Z-Value	p-Value
Brown et al. 2020	TF-CBT	Depression	0,504	0,426	0,181	-0,331	1,339	1,183	0,237
Cohen et al. 1998; 2005	TF-CBT	Depression	0,422	0,175	0,031	0,078	0,766	2,406	0,016
Cohen, Deblinger et al. 2004; Deblinger et al. 2006	TF-CBT	Depression	0,865	0,141	0,020	0,589	1,141	6,140	0,000
Deblinger et al., 1996; 1999	TF-CBT (child only)	Depression	1,111	0,297	0,088	0,529	1,692	3,741	0,000
Deblinger et al., 1996; 1999	TF-CBT (mother and child)	Depression	0,702	0,271	0,073	0,170	1,233	2,589	0,010
Goldbeck et al. 2016; Tutus et al. 2017	TF-CBT	Depression	0,672	0,112	0,012	0,454	0,891	6,027	0,000
Jensen et al. 2014, 2017	TF-CBT	Depression	1,432	0,205	0,042	1,030	1,834	6,981	0,000
Nixon et al. 2012; 2017	TF-CBT	Depression	1,787	0,386	0,149	1,030	2,543	4,631	0,000
O'Donnel et al. 2014	TF-CBT group	Depression	1,638	0,214	0,046	1,220	2,057	7,674	0,000
			0,994	0,152	0,023	0,697	1,292	6,552	0,000

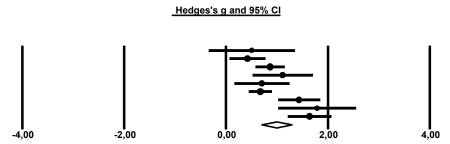

Table 10.

Anxiety Pre-FU III Within-Group Effect Size.

Study name	Condition	Outcome	Hedges's g	Standard error	Variance	Lower limit	Upper limit	Z-Value	p-Value
Brown et al. 2020	TF-CBT	Anxiety	-0,034	0,362	0,131	-0,743	0,675	-0,094	0,925
Cohen et al. 1998; 2005	TF-CBT	Anxiety	0,575	0,182	0,033	0,219	0,932	3,167	0,002
Cohen, Deblinger et al. 2004; Deblinger et al. 2006	TF-CBT	Anxiety	1,214	0,159	0,025	0,903	1,525	7,655	0,000
Goldbeck et al. 2016; Tutus et al. 2017	TF-CBT	Anxiety	0,754	0,128	0,016	0,504	1,005	5,902	0,000
Jensen et al. 2014, 2017	TF-CBT	Anxiety	0,996	0,181	0,033	0,642	1,350	5,514	0,000
Nixon et al. 2012; 2017	TF-CBT	Anxiety	2,617	0,451	0,203	1,734	3,500	5,807	0,000
			0,949	0,202	0,041	0,552	1,345	4,689	0,000

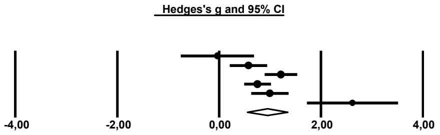

Table 11.

Grief Pre-FU III Within-Group Effect Size.

Study name	Condition	Outcome	Hedges's g	Standard error	Variance	Lower limit	Upper limit	Z-Value	p-Value
Brown et al. 2020	TF-CBT	Grief	0,173	0,370	0,137	-0,551	0,898	0,469	0,639
Dorsey et al. 2020	TF-CBT group (KR)	Grief	1,683	0,212	0,045	1,268	2,098	7,947	0,000
Dorsey et al. 2020	TF-CBT group (KU)	Grief	1,682	0,173	0,030	1,343	2,021	9,713	0,000
Dorsey et al. 2020	TF-CBT group (TR)	Grief	1,976	0,234	0,055	1,517	2,435	8,433	0,000
Dorsey et al. 2020	TF-CBT group (TU)	Grief	2,205	0,207	0,043	1,800	2,610	10,673	0,000
O'Donnel et al. 2014	TF-CBT group	Grief	2,352	0,271	0,074	1,820	2,884	8,670	0,000
			1,730	0,227	0,052	1,285	2,175	7,621	0,000

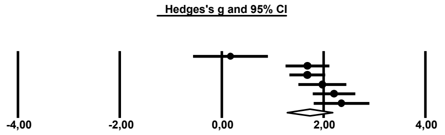

KR = Kenia Rural; KU = Kenia Urban; TR = Tanzania Rural; TU = Tanzania Urban.

Table 12.

PTSS FU II-FU III Within-Group Effect Size.

Study name	Condition	Outcome	Hedges's g	Standard error	Variance	Lower limit	Upper limit	Z-Value	p-Value
Brown et al. 2020	TF-CBT	PTSS	0,115	0,365	0,133	-0,600	0,831	0,316	0,752
Cohen et al. 1998; 2005	TF-CBT	PTSS	0,118	0,168	0,028	-0,212	0,449	0,703	0,482
Cohen, Deblinger et al. 2004; Deblinger et al. 2006	TF-CBT	PTSS	0,220	0,123	0,015	-0,021	0,461	1,791	0,073
Deblinger et al., 1996; 1999	TF-CBT (child only)	PTSS	0,296	0,235	0,055	-0,166	0,757	1,256	0,209
Deblinger et al., 1996; 1999	TF-CBT (mother and child)	PTSS	0,153	0,242	0,059	-0,322	0,628	0,632	0,527
Goldbeck et al. 2016; Tutus et al. 2017	TF-CBT	PTSS	0,164	0,115	0,013	-0,062	0,389	1,422	0,155
Nixon et al. 2012; 2017	TF-CBT	PTSS	0,476	0,268	0,072	-0,050	1,002	1,774	0,076
			0,199	0,065	0,004	0,071	0,327	3,045	0,002

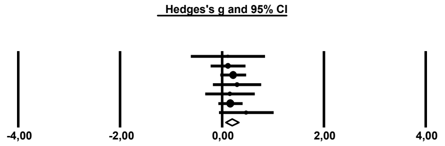

Table 13.

Depression FU II-FU III Within-Group Effect Size.

Study name	Condition	Outcome	Hedges's g	Standard error	Variance	Lower limit	Upper limit	Z-Value	p-Value
Brown et al. 2020	TF-CBT	Depression	0,100	0,364	0,133	-0,614	0,813	0,273	0,785
Cohen et al. 1998; 2005	TF-CBT	Depression	0,045	0,168	0,028	-0,284	0,374	0,268	0,789
Cohen, Deblinger et al. 2004; Deblinger et al. 2006	TF-CBT	Depression	0,014	0,120	0,014	-0,221	0,249	0,119	0,905
Deblinger et al., 1996; 1999	TF-CBT (child only)	Depression	0,013	0,230	0,053	-0,437	0,464	0,059	0,953
Deblinger et al., 1996; 1999	TF-CBT (mother and child)	Depression	0,124	0,242	0,058	-0,350	0,597	0,511	0,609
Goldbeck et al. 2016; Tutus et al. 2017	TF-CBT	Depression	0,093	0,101	0,010	-0,104	0,291	0,927	0,354
Nixon et al. 2012; 2017	TF-CBT	Depression	0,534	0,272	0,074	0,000	1,067	1,960	0,050
			0,085	0,062	0,004	-0,037	0,206	1,369	0,171

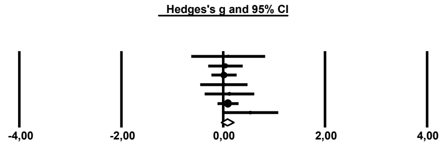

Table 14.

Anxiety FU II-FU III Within-Group Effect Size.

Study name	Condition	Outcome	Hedges's g	Standard error	Variance	Lower limit	Upper limit	Z-Value	p-Value
Brown et al. 2020	TF-CBT	Anxiety	-0,512	0,428	0,183	-1,350	0,327	-1,196	0,232
Cohen et al. 1998; 2005	TF-CBT	Anxiety	0,030	0,168	0,028	-0,299	0,359	0,178	0,859
Cohen, Deblinger et al. 2004; Deblinger et al. 2006	TF-CBT	Anxiety	0,149	0,121	0,015	-0,087	0,385	1,237	0,216
Goldbeck et al. 2016; Tutus et al. 2017	TF-CBT	Anxiety	0,126	0,113	0,013	-0,096	0,347	1,114	0,265
Nixon et al. 2012; 2017	TF-CBT	Anxiety	0,400	0,264	0,070	-0,117	0,917	1,515	0,130
			0,119	0,070	0,005	-0,019	0,257	1,695	0,090

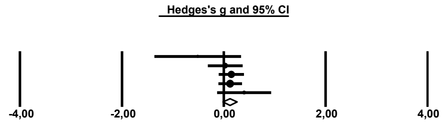

Table 15.

PTSS Pre-Post Proxy-Reported Within-Group Effect Size.

Study name	Condition	Outcome	Hedges's g	Standard error	Variance	Lower limit	Upper limit	Z-Value	p-Value
Allen et al., 2021	TF-CBT	PTSS	1,554	0,398	0,158	0,775	2,334	3,908	0,000
Allen et al., 2021	TF-CBT (+ AAT)	PTSS	1,145	0,332	0,110	0,494	1,796	3,448	0,001
Amaya-Jackson et al. 2018	TF-CBT	PTSS	0,874	0,103	0,011	0,672	1,075	8,505	0,000
Cohen et al. 2006	TF-CBT	PTSS	0,512	0,176	0,031	0,167	0,858	2,907	0,004
Cohen, Mannarino et al., 2004	TF-CBT	PTSS	1,654	0,323	0,104	1,021	2,288	5,119	0,000
Davidson et al. 2018	TF-CBT	PTSS	0,253	0,368	0,135	-0,468	0,973	0,687	0,492
Davidson et al. 2018	TF-CBT (+ tablet)	PTSS	0,550	0,273	0,075	0,014	1,086	2,013	0,044
Dorsey et al. 2014	TF-CBT	PTSS	0,963	0,238	0,056	0,497	1,429	4,052	0,000
Dorsey et al. 2020	TF-CBT group (KR)	PTSS	1,969	0,234	0,055	1,511	2,428	8,423	0,000
Dorsey et al. 2020	TF-CBT group (KU)	PTSS	2,054	0,197	0,039	1,669	2,439	10,445	0,000
Dorsey et al. 2020	TF-CBT group (TR)	PTSS	2,307	0,261	0,068	1,795	2,818	8,834	0,000
Dorsey et al. 2020	TF-CBT group (TU)	PTSS	2,125	0,201	0,041	1,730	2,519	10,556	0,000
Goldbeck et al. 2016; Tutus et al. 2017	TF-CBT	PTSS	0,740	0,151	0,023	0,444	1,036	4,902	0,000
Jaberghaderi et al. 2004	TF-CBT	PTSS	1,087	0,481	0,231	0,145	2,029	2,262	0,024
Kirsch et al., 2011	TF-CBT	PTSS	0,559	0,265	0,070	0,041	1,078	2,114	0,035
Konanur et al. 2015	TF-CBT	PTSS	0,281	0,154	0,024	-0,021	0,583	1,821	0,069
Lang et al. 2015	TF-CBT	PTSS	0,543	0,079	0,006	0,387	0,698	6,840	0,000
Langley et al. 2015	TF-CBT group (Bounce Back, delayed)	PTSS	0,770	0,192	0,037	0,394	1,146	4,015	0,000
Langley et al. 2015	TF-CBT group (Bounce Back, immediate)	PTSS	0,602	0,197	0,039	0,215	0,989	3,049	0,002
O'Donnel et al. 2014	TF-CBT group	PTSS	1,200	0,184	0,034	0,839	1,561	6,519	0,000
Santiago et al. 2018	TF-CBT group (Bounce Back, delayed)	PTSS	-0,109	0,187	0,035	-0,476	0,258	-0,581	0,561
Santiago et al. 2018	TF-CBT group (Bounce Back, immediate)	PTSS	0,200	0,214	0,046	-0,220	0,620	0,933	0,351
Schottelkorb et al. 2012	TF-CBT	PTSS	0,040	0,305	0,093	-0,558	0,638	0,131	0,896
Stewart et al. 2017	TF-CBT	PTSS	1,640	0,399	0,159	0,858	2,422	4,109	0,000
Stewart et al. 2020	TF-CBT (DSM-IV)	PTSS	1,479	0,236	0,055	1,018	1,941	6,280	0,000
Stewart et al. 2020	TF-CBT (DSM-V)	PTSS	1,407	0,334	0,112	0,752	2,061	4,212	0,000
Unterhitzenberger et al. 2019	TF-CBT	PTSS	1,367	0,294	0,087	0,790	1,943	4,647	0,000
Espeleta et al. 2021	TF-CBT	PTSS	1,091	0,127	0,016	0,842	1,339	8,591	0,000
			1,014	0,115	0,013	0,789	1,239	8,838	0,000

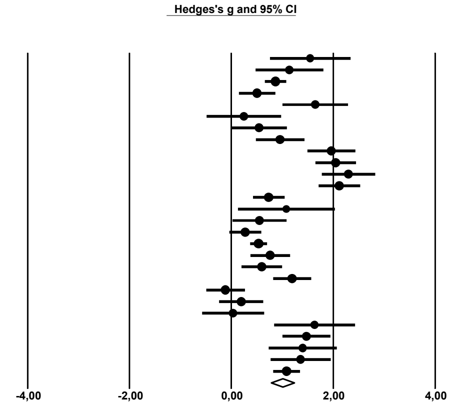

AAT = Animal-Assisted Therapy; KR = Kenia Rural; KU = Kenia Urban; TR = Tanzania Rural; TU = Tanzania Urban.

Table 16.

Depression Pre-Post Proxy-Reported Within-Group Effect Size.

Study name	Condition	Outcome	Hedges's g	Standard error	Variance	Lower limit	Upper limit	Z-Value	p-Value
Diehle et al. 2015	TF-CBT	Depression	0,876	0,367	0,135	0,157	1,596	2,387	0,017
Lang et al. 2015	TF-CBT	Depression	0,546	0,087	0,008	0,375	0,717	6,246	0,000
Langley et al. 2015	TF-CBT group (Bounce Back, delayed)	Depression	0,414	0,175	0,031	0,071	0,756	2,364	0,018
Langley et al. 2015	TF-CBT group (Bounce Back, immediate)	Depression	0,296	0,185	0,034	-0,067	0,659	1,598	0,110
O'Donnel et al. 2014	TF-CBT group	Depression	0,849	0,158	0,025	0,539	1,159	5,363	0,000
Santiago et al. 2018	TF-CBT group (Bounce Back, delayed)	Depression	0,456	0,197	0,039	0,071	0,842	2,318	0,020
Santiago et al. 2018	TF-CBT group (Bounce Back, immediate)	Depression	0,207	0,215	0,046	-0,214	0,627	0,964	0,335
Stewart et al. 2017	TF-CBT	Depression	0,717	0,286	0,082	0,157	1,277	2,509	0,012
Stewart et al. 2020	TF-CBT	Depression	0,866	0,161	0,026	0,551	1,182	5,384	0,000
Unterhitzenberger et al. 2019	TF-CBT	Depression	1,173	0,274	0,075	0,636	1,710	4,279	0,000
			0,607	0,085	0,007	0,442	0,773	7,182	0,000

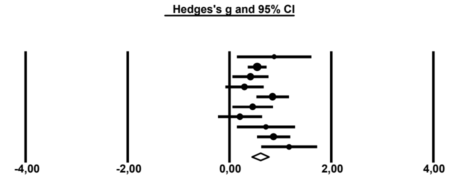

Table 17.

Anxiety Pre-Post Proxy-Reported Within-Group Effect Size.

Study name	Condition	Outcome	Hedges's g	Standard error	Variance	Lower limit	Upper limit	Z-Value	p-Value
Diehle et al. 2015	TF-CBT	Anxiety	0,467	0,337	0,113	-0,193	1,127	1,387	0,166
Goldbeck et al. 2016; Tutus et al. 2017	TF-CBT	Anxiety	0,524	0,105	0,011	0,319	0,730	4,999	0,000
Stewart et al. 2017	TF-CBT	Anxiety	0,556	0,273	0,074	0,022	1,090	2,039	0,041
			0,524	0,094	0,009	0,339	0,708	5,571	0,000

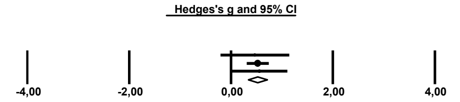

Table 18.

PTSS Pre-FU III Proxy-Reported Within-Group Effect Size.

Study name	Condition	Outcome	Hedges's g	Standard error	Variance	Lower limit	Upper limit	Z-Value	p-Value
Dorsey et al. 2020	TF-CBT group (KR)	PTSS	2,952	0,316	0,100	2,332	3,572	9,335	0,000
Dorsey et al. 2020	TF-CBT group (KU)	PTSS	2,008	0,194	0,037	1,628	2,387	10,368	0,000
Dorsey et al. 2020	TF-CBT group (TR)	PTSS	2,219	0,254	0,064	1,721	2,717	8,739	0,000
Dorsey et al. 2020	TF-CBT group (TU)	PTSS	2,720	0,242	0,059	2,246	3,194	11,242	0,000
Goldbeck et al. 2016; Tutus et al. 2017	TF-CBT	PTSS	0,811	0,147	0,022	0,522	1,099	5,504	0,000
O'Donnel et al. 2014	TF-CBT group	PTSS	1,520	0,205	0,042	1,119	1,921	7,426	0,000
			2,017	0,344	0,118	1,342	2,691	5,863	0,000

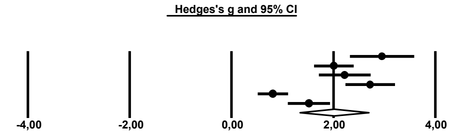

KR = Kenia Rural; KU = Kenia Urban; TR = Tanzania Rural; TU = Tanzania Urban.

Table 19.

PTSS FU III Between-Group Effect Size.

Study name	Condition	Outcome	Hedges's g	Standard error	Variance	Lower limit	Upper limit	Z-Value	p-Value
Brown et al. 2020	TF-CBT	PTSS	-0,698	0,659	0,434	-1,989	0,593	-1,060	0,289
Cohen et al. 1998; 2005	TF-CBT	PTSS	0,472	0,222	0,049	0,037	0,907	2,128	0,033
Cohen, Deblinger et al. 2004; Deblinger et al. 2006	TF-CBT	PTSS	0,317	0,162	0,026	-0,000	0,635	1,957	0,050
Deblinger et al., 1996; 1999	TF-CBT (child only)	PTSS	0,217	0,307	0,094	-0,385	0,820	0,707	0,480
Deblinger et al., 1996; 1999	TF-CBT (mother and child)	PTSS	0,468	0,318	0,101	-0,156	1,092	1,470	0,142
Dorsey et al. 2020	TF-CBT group (KR)	PTSS	0,946	0,185	0,034	0,583	1,309	5,103	0,000
Dorsey et al. 2020	TF-CBT group (KU)	PTSS	0,747	0,149	0,022	0,456	1,039	5,025	0,000
Dorsey et al. 2020	TF-CBT group (TR)	PTSS	-0,066	0,176	0,031	-0,411	0,278	-0,377	0,706
Dorsey et al. 2020	TF-CBT group (TU)	PTSS	0,165	0,144	0,021	-0,117	0,447	1,145	0,252
Jensen et al. 2014, 2017	TF-CBT	PTSS	0,169	0,201	0,040	-0,225	0,563	0,842	0,400
Nixon et al. 2012; 2017	TF-CBT	PTSS	0,175	0,340	0,116	-0,493	0,842	0,513	0,608
			0,345	0,111	0,012	0,127	0,563	3,108	0,002

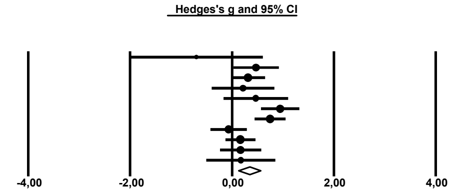

KR = Kenia Rural; KU = Kenia Urban; TR = Tanzania Rural; TU = Tanzania Urban.

Table 20.

Depression FU III Between-Group Effect Size.

Study name	Condition	Outcome	Hedges's g	Standard error	Variance	Lower limit	Upper limit	Z-Value	p-Value
Brown et al. 2020	TF-CBT	Depression	-0,297	0,627	0,393	-1,525	0,932	-0,474	0,636
Cohen et al. 1998; 2005	TF-CBT	Depression	0,176	0,219	0,048	-0,254	0,605	0,802	0,423
Cohen, Deblinger et al. 2004; Deblinger et al. 2006	TF-CBT	Depression	0,174	0,160	0,026	-0,141	0,488	1,082	0,279
Deblinger et al., 1996; 1999	TF-CBT (child only)	Depression	0,196	0,307	0,094	-0,406	0,798	0,639	0,523
Deblinger et al., 1996; 1999	TF-CBT (mother and child)	Depression	-0,188	0,315	0,099	-0,804	0,429	-0,597	0,551
Jensen et al. 2014, 2017	TF-CBT	Depression	0,200	0,204	0,042	-0,200	0,600	0,980	0,327
Nixon et al. 2012; 2017	TF-CBT	Depression	0,137	0,340	0,116	-0,530	0,804	0,403	0,687
			0,137	0,093	0,009	-0,045	0,319	1,471	0,141

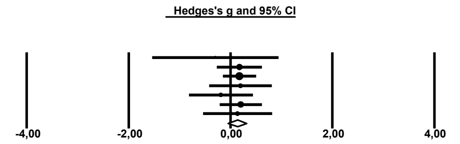

Table 21.

Anxiety FU III Between-Group Effect Size.

Study name	Condition	Outcome	Hedges's g	Standard error	Variance	Lower limit	Upper limit	Z-Value	p-Value
			Statistics for each study						
Brown et al. 2020	TF-CBT	Anxiety	-1,308	0,689	0,474	-2,658	0,041	-1,900	0,057
Cohen et al. 1998; 2005	TF-CBT	Anxiety	0,126	0,219	0,048	-0,304	0,555	0,573	0,566
Cohen, Deblinger et al. 2004; Deblinger et al. 2006	TF-CBT	Anxiety	0,277	0,161	0,026	-0,038	0,593	1,724	0,085
Jensen et al. 2014, 2017	TF-CBT	Anxiety	0,202	0,202	0,041	-0,195	0,599	0,997	0,319
Nixon et al. 2012; 2017	TF-CBT	Anxiety	0,190	0,341	0,116	-0,478	0,857	0,557	0,577
			0,163	0,123	0,015	-0,078	0,404	1,323	0,186

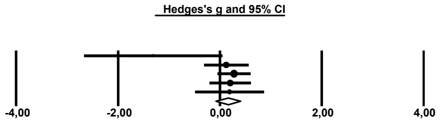

Table 22.

Grief FU III Between-Group Effect Size.

Study name	Condition	Outcome	Hedges's g	Standard error	Variance	Lower limit	Upper limit	Z-Value	p-Value
			Statistics for each study						
Brown et al. 2020	TF-CBT	Grief	-0,694	0,658	0,433	-1,985	0,596	-1,055	0,292
Dorsey et al. 2020	TF-CBT group (KU)	Grief	0,607	0,147	0,022	0,318	0,895	4,125	0,000
Dorsey et al. 2020	TF-CBT group (TR)	Grief	-0,036	0,176	0,031	-0,380	0,309	-0,202	0,840
Dorsey et al. 2020	TF-CBT group (TU)	Grief	0,216	0,144	0,021	-0,067	0,498	1,496	0,135
Dorsey et al. 2020	TF-CBT group (KR)	Grief	0,775	0,182	0,033	0,418	1,132	4,251	0,000
			0,327	0,177	0,031	-0,020	0,673	1,849	0,064

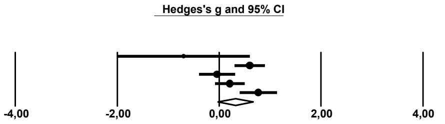

Table 23.

PTSS Post-Treatment Proxy-Reported Between-Group Effect Size.

Study name	Condition	Outcome	Hedges's g	Standard error	Variance	Lower limit	Upper limit	Z-Value	p-Value
			Statistics for each study						
Dorsey et al. 2020	TF-CBT group (KR)	PTSS	1,677	0,205	0,042	1,276	2,078	8,196	0,000
Dorsey et al. 2020	TF-CBT group (KU)	PTSS	0,764	0,149	0,022	0,472	1,056	5,128	0,000
Dorsey et al. 2020	TF-CBT group (TR)	PTSS	0,369	0,177	0,031	0,021	0,716	2,080	0,038
Dorsey et al. 2020	TF-CBT group (TU)	PTSS	0,313	0,145	0,021	0,030	0,597	2,164	0,030
Goldbeck et al. 2016; Tutus et al. 2017	TF-CBT	PTSS	0,420	0,163	0,027	0,100	0,740	2,574	0,010
Langley et al. 2015	TF-CBT group (Bounce Back, immediate)	PTSS	0,908	0,247	0,061	0,424	1,392	3,678	0,000
Santiago et al. 2018	TF-CBT group (Bounce Back, immediate)	PTSS	-0,409	0,276	0,076	-0,951	0,132	-1,481	0,139
Schottelkorb et al. 2012	TF-CBT	PTSS	-0,242	0,397	0,158	-1,021	0,536	-0,610	0,542
Jaberghaderi et al. 2004	TF-CBT	PTSS	-0,179	0,502	0,252	-1,162	0,804	-0,356	0,722
			0,461	0,189	0,036	0,091	0,831	2,445	0,014

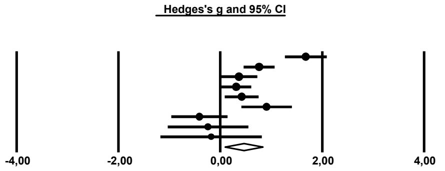

KR = Kenia Rural; KU = Kenia Urban; TR = Tanzania Rural; TU = Tanzania Urban.

Table 24.

Depression Post-Treatment Proxy-Reported Between-Group Effect Size.

Study name	Condition	Outcome	Hedges's g	Standard error	Variance	Lower limit	Upper limit	Z-Value	p-Value
			Statistics for each study						
Langley et al. 2015	TF-CBT group (Bounce Back, immediate)	Depression	0,588	0,240	0,058	0,118	1,058	2,451	0,014
Santiago et al. 2018	TF-CBT group (Bounce Back, immediate)	Depression	0,202	0,274	0,075	-0,335	0,739	0,737	0,461
Diehle et al. 2015	TF-CBT	Depression	-0,315	0,433	0,188	-1,165	0,534	-0,728	0,467
			0,256	0,233	0,054	-0,202	0,713	1,095	0,273

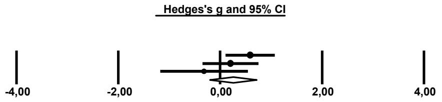

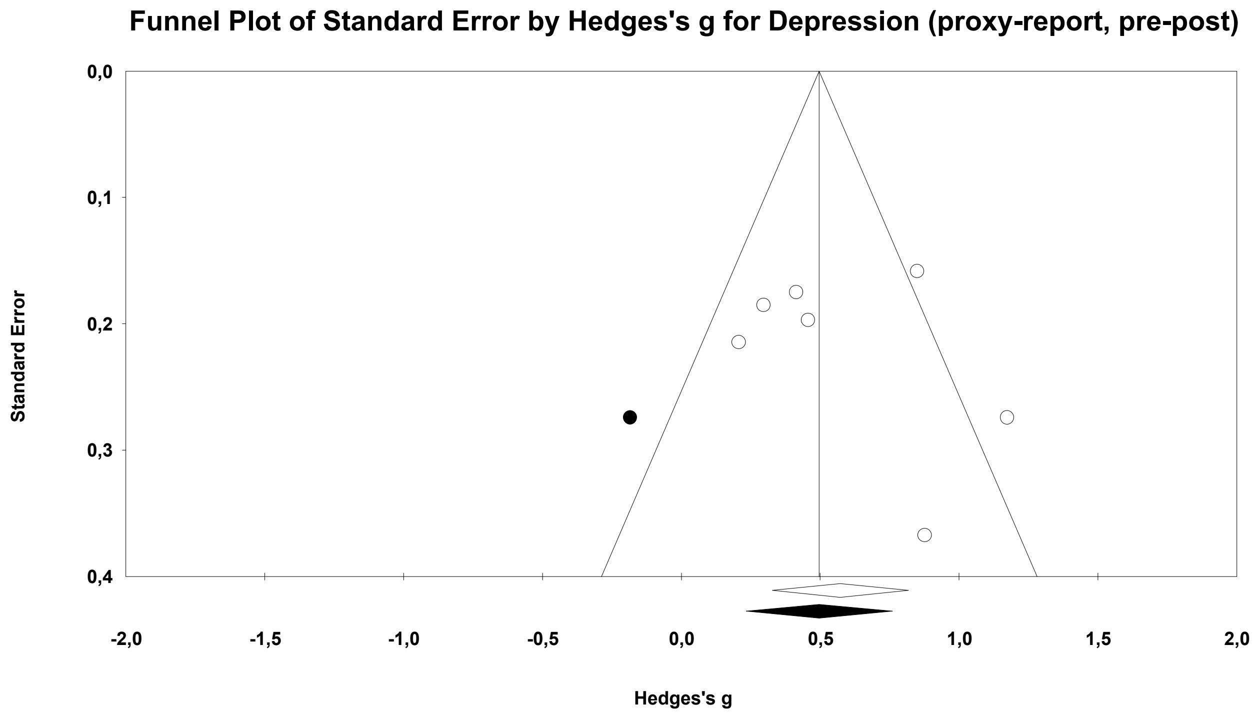

Note. Black circle represents imputed study.

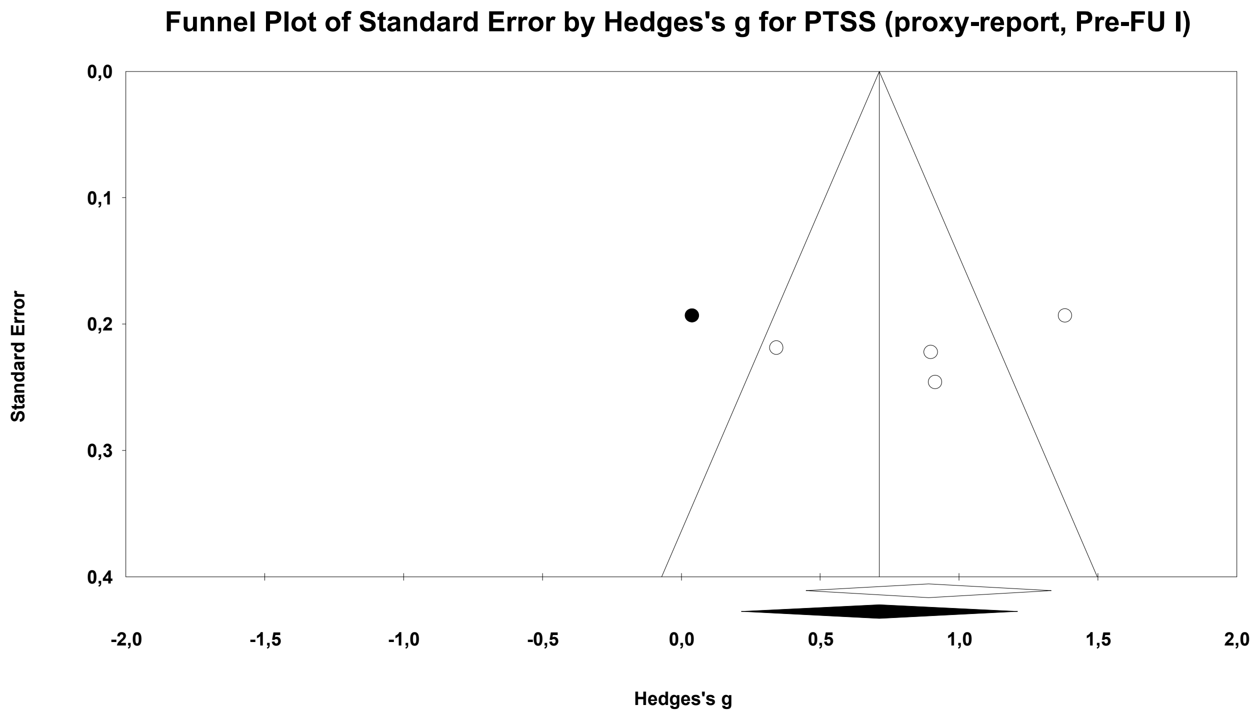

Note. Black circle represents imputed study.

Funnel Plot of Standard Error by Hedges's g for PTSS (all studies, pre-FU III)

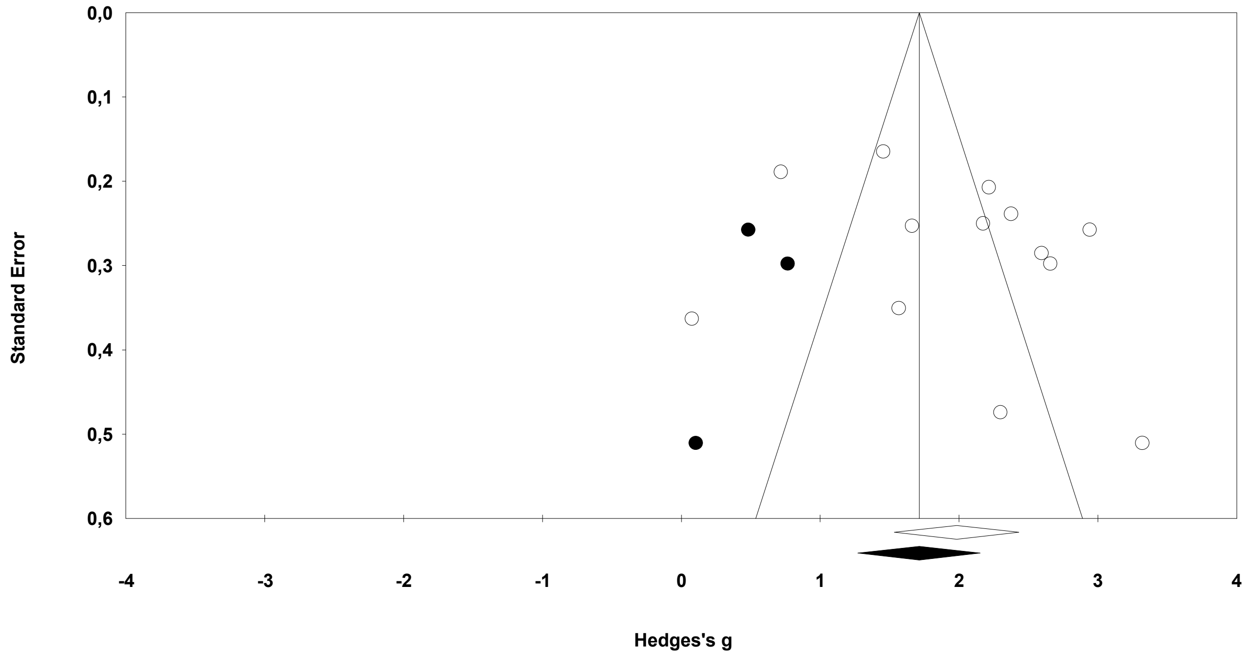

Note. Black circles represent imputed studies.

Funnel Plot of Standard Error by Hedges's g for PTSS (RCTs only, Pre-FU III)

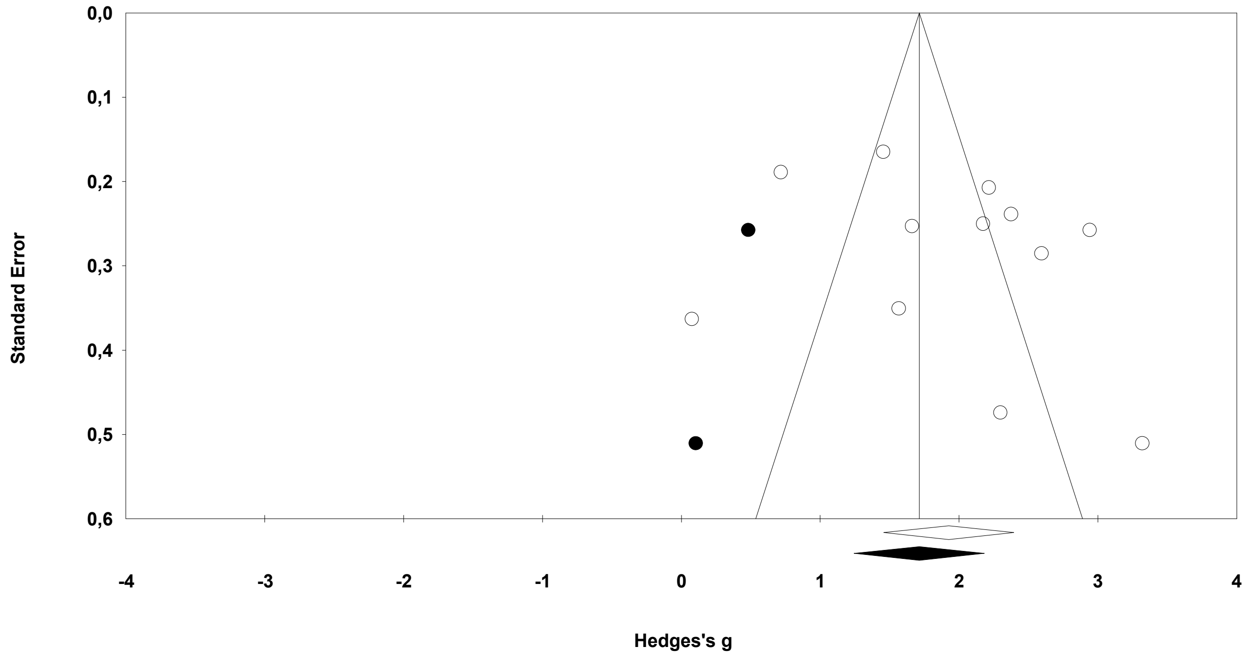

Note. Black circles represent imputed studies.

Funnel Plot of Standard Error by Hedges's g for PTSS (Effectiveness RCTs, pre-FU III)

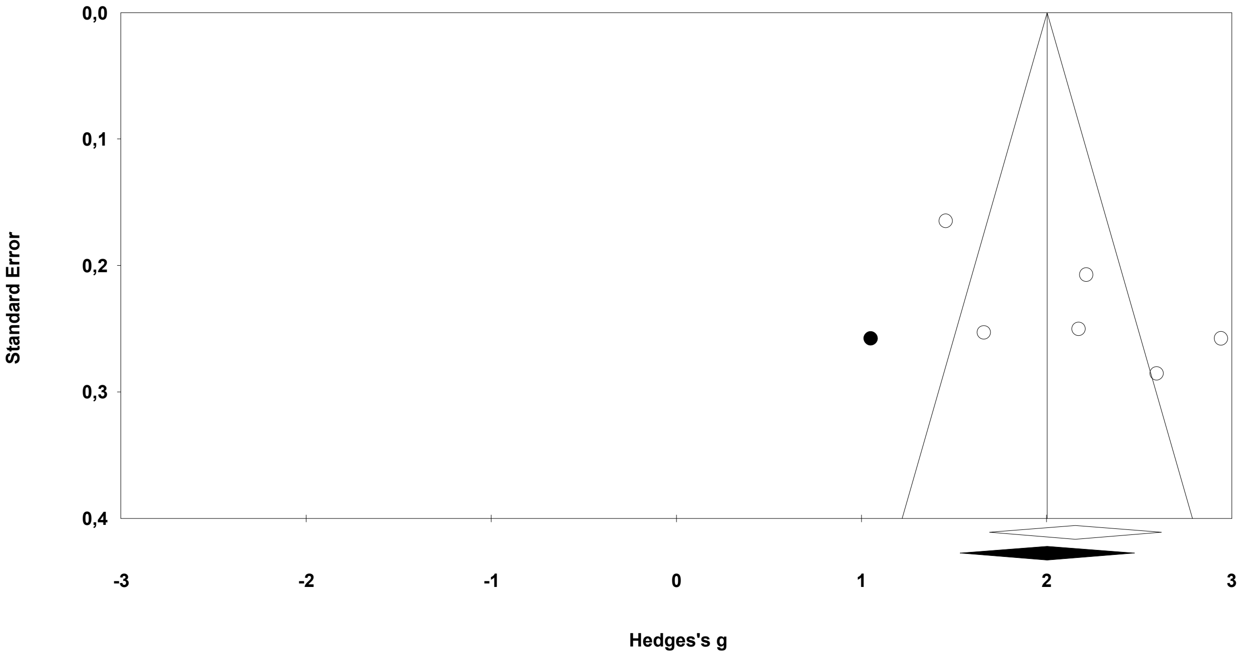

Note. Black circle represents imputed study.

Funnel Plot of Standard Error by Hedges's g for Grief (all studies, pre-FU III)

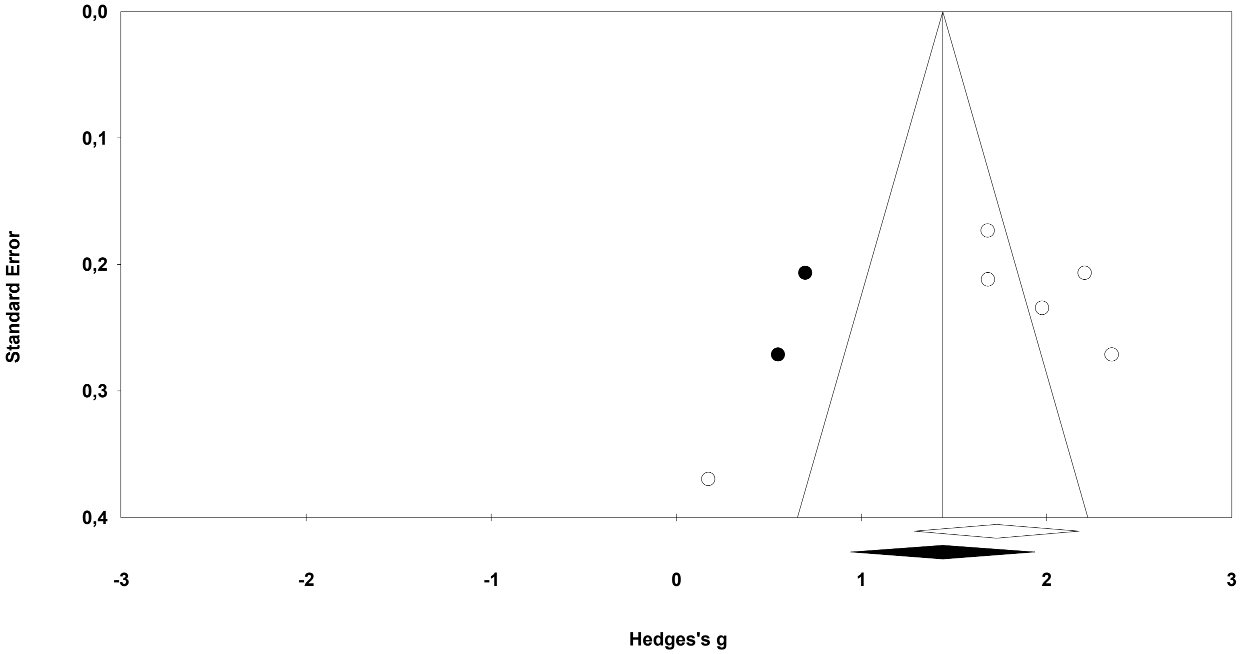

Note. Black circles represent imputed studies.

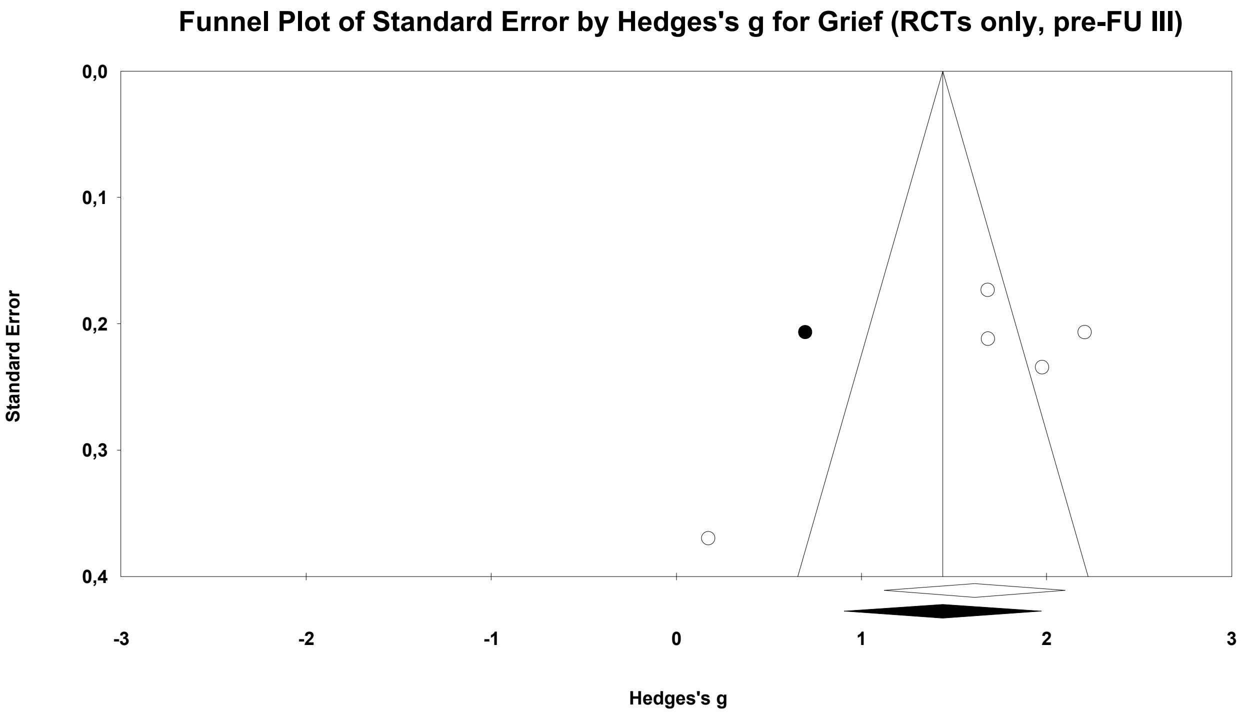

Note. Black circle represents imputed study.

Table 25

Pre-FU I Within-Group Effect Sizes for PTSS, Depression and Anxiety.

Outcome	Sample		n	g	95% CI	SE	z	Q	I²	Duval and Tweedie
PTSS	All eligible studies		9	(1.79)<br>1.63[a]	(1.36 - 2.21)<br>1.22 - 2.04	(0.22)	(8.25***)	25.89**	69.10	2
	Individual		4	1.71[b]	1.32 - 2.11	.20	8.55***	1.55	0	0
	Group		5	(1.87)<br>1.70	(1.15-2.59)<br>1.04 - 2.36	(.37)	(5.07***)	24.26***	83.51	1
	RCTs only		8	(1.73)<br>1.42	(1.27-2.19)<br>.98 - 1.87	(.26)	(7.36***)	21.08**	66.80	3
		Efficacy	4	1.71[b]	1.32 - 2.11	.20	8.55***	1.55	0	0
		Effectiveness	4	1.80	.91 - 2.70	.46	3.95***	18.23***	83.54	0
		Individual	4	1.71[b]	1.32 - 2.11	.20	8.55***	1.55	0	0
		Group	4	1.80	.91 - 2.70	.46	3.95***	18.23***	83.54	0
Depression	All eligible studies		6	.89	.54 - 1.24	.18	4.95***	16.94**	70.49	0
	Individual		3	.78[b]	.48 - 1.07	.15	5.10***	.70	0	0
	Group		3	.98	.31 - 1.65	.34	2.85**	14.90**	86.57	0
	RCTs only		5	.73[b]	.51 - .94	.11	6.62***	4.21	5.03	0
Anxiety	All eligible studies		4	(.62[b,c])<br>.38[a,b,c]	(.37 - .88)<br>.17 - .59	(.13)	(4.79***)	5.00	40.01	2
	RCTs only		4	(.62[b,c])<br>.38[a,b,c]	(.37 - .88)<br>.17 - .59	(.13)	(4.79***)	5.00	40.01	2

Note. High risk of bias studies are excluded; n = number of included TF-CBT conditions. * $p < .05$; ** $p < .01$; *** $p < .001$.

[a] Recalculated with imputed studies (trim and fill method according to Duval and Tweedie).

[b] Fixed model assumed due to non-significant Q-value.

[c] Analysis includes high risk of bias studies (calculation otherwise not possible due to low number of studies).

Table 26

Pre-FU II Within-Group Effect Sizes for PTSS, Depression and Anxiety.

Outcome	Sample	n	g	95% CI	SE	z	Q	I^2	Duval and Tweedie
PTSS	All eligible studies	9	1.65	1.07 - 2.24	.30	5.54***	82.41***	90.29	0
	Individual	8	1.43	.89 - 1.98	.28	5.12***	62.28***	88.76	0
	RCTs only	8	(1.79)<br>1.56[a]	(1.13 - 2.45)<br>.91 - 2.22	(.34)	(5.33***)	76.66***	90.87	1
Depression	All eligible studies	8	(.63[b])<br>.60[a,b]	(.50 - .76)<br>.48 - .73	(.07)	(9.40***)	11.96	41.49	1
	Individual	8	(.63[b])<br>.60[a,b]	(.50 - .76)<br>.48 - .73	(.07)	(9.40***)	11.96	41.49	1
	RCTs only	7	(.67[b])<br>.63[a,b]	(.53 - .81)<br>.49 - .76	(.07)	(9.46***)	8.98	33.18	2
Anxiety	All eligible studies	5	.82	.52 - 1.11	.15	5.45***	10.36*	61.39	0
	Individual	5	.82	.52 - 1.11	.15	5.45***	10.36*	61.39	0
	RCTs only	5	.82	.52 - 1.11	.15	5.45***	10.36*	61.39	0

Note. High risk of bias studies are excluded; n = number of included TF-CBT conditions. * $p < .05$; ** $p < .01$; *** $p < .001$.

[a] Recalculated with imputed studies (trim and fill method according to Duval and Tweedie).

[b] Fixed model assumed due to non-significant Q-value.

Table 27

Pre-Post Within-Group Effect Sizes for PTSS, Depression, Anxiety and Grief.

Outcome	Sample	n	g	95% CI	SE	z	Q	I^2	Duval and Tweedie
PTSS	FU I	9	1.60	1.23 - 1.97	.19	8.39***	25.11**	68.13	0
	FU II	9	(1.18)	(.73 - 1.62)	(.23)	(5.20***)	83.93***	90.47	2
			.92[a]	.51 - 1.34					
	FU III	13	(1.43)	(1.00 - 1.86)	(.22)	(6.47***)	216.91***	94.47	1
			1.38[a]	.97 - 1.80					
Depression	FU I	6	.67	.41 - .93	.14	4.98***	11.17*	55.23	0
	FU II	8	(.43[b)]	.31-.55	.06	7.15***	8.66	19.18	2
			.41[a,b]	.30 - .52					
	FU III	9	.71	.41 - 1.01	.15	4.69***	46.58***	82.82	0
Anxiety	FU I	4	.43[b,c]	.19 - .67	.12	3.56***	1.66	0	0
	FU II	5	.47	.18-.77	.15	3.15**	13.92**	71.27	0
	FU III	6	.55	.27 - .83	.15	3.78***	20.27**	75.33	0
Grief	FU III	6	1.42[b]	1.27 - 1.57	.08	18.15***	4.07	0	0

Note. High risk of bias studies are excluded; n = number of included TF-CBT conditions; Individual = TF-CBT conducted in an individual treatment setting; Group = TF-CBT conducted in a group setting. * $p < .05$; ** $p < .01$; *** $p < .001$.

[a] Recalculated with imputed studies (trim and fill method according to Duval and Tweedie).

[b] Fixed model assumed due to non-significant Q-value.

[c] Analysis includes high risk of bias studies (calculation otherwise not possible due to low number of studies).

Table 28

Pre-Post Within-Group Effect Sizes for Proxy-Reported PTSS and Depression.

Outcome	Sample	n	g	95% CI	SE	z	Q	I²	Duval and Tweedie
PTSS	FU I	4	.75	.31 - 1.18	.22	3.34**	13.90**	78.42	0
	FU II	3	.75[a]	.23 - 1.27	.27	2.81**	11.92**	83.23	0
	FU III	6	1.72	1.17 - 2.26	.28	6.18***	58.74***	91.49	0
Depression	FU I	3	.47	.05 - .88	.21	2.20*	7.92*	74.73	0

Note. High risk of bias studies are excluded; n = number of included TF-CBT conditions; Individual = TF-CBT conducted in an individual treatment setting; Group = TF-CBT conducted in a group setting. * $p < .05$; ** $p < .01$; *** $p < .001$.

[a] Analysis includes high risk of bias studies (calculation otherwise not possible due to low number of studies).

Table 29

Post-treatment Between-Group Effect Sizes for PTSS, Depression, Anxiety and Grief.

Assessment point	Outcome	Sample	n	g	95% CI	SE	z	Q	I^2	Duval and Tweedie
FU I	PTSS	Any control	4	(.69[a,b])<br>.58[a,b,c]	(.34 - 1.04)<br>.26 - .90	(.18)	(3.84***)	3.33	9.96	1
	Depression	Any control	4	(.24[a,b])<br>.22[a,b,c]	(-.10 - .58)<br>-.09 - .53	(.17)	(1.37)	.09	0	1
FU II	PTSS	TAU/AT	7	.34[a]	.15 - .53	.10	3.58***	7.98	24.83	0
	Depression	TAU/AT	6	.32[a]	.13 - .52	.10	3.24**	2.98	0	0
	Anxiety	TAU/AT	4	.20[a]	-.02 - .42	.11	1.78	6.89	56.44	0
FU III	PTSS	TAU/AT	11	.37	.16 - .59	.11	3.39**	29.80**	66.45	0
		Efficacy	6	.39[a]	.19 - .59	.10	3.87***	5.80	13.84	0
		Effectiveness	5	.42	.06 - .77	.18	2.29*	24.00***	83.33	0
		Individual	7	.42[a]	.24 - .59	.09	4.70***	6.06	1.05	0
		Group	4	.40	-.05 - .84	.23	1.75	23.62***	87.30	0
	Depression	TAU/AT	7	.37[a]	.20-.55	.09	4.25***	4.09	0	0
	Anxiety	TAU/AT	5	.23[a]	.04 - .41	.10	2.35*	7.09	43.59	0
	Grief	TAU/AT	5	.30	-06 - .67	.19	1.63	19.58**	79.57	0

Note. High risk of bias studies are excluded; n = number of included comparisons; PTSS = Posttraumatic Stress Symptoms; TAU/AT = Treatment as usual/active treatment control conditions; WL = Wait-list control conditions; Effectiveness = Effectiveness RCTs only; Efficacy = Efficacy RCTs only; Individual = RCTs conducted in an individual treatment setting; Group = RCTs conducted in a group setting. * $p < .05$; ** $p < .01$; *** $p < .001$.

[a] Fixed model assumed due to non-significant Q-value.

[b] Analysis includes high risk of bias studies (calculation otherwise not possible due to low number of studies).

[c] Recalculated with imputed studies (trim and fill method according to Duval and Tweedie).

Intention-to-treat

Unique ID	Experimental	Comparator	Outcome	D1	D2	D3	D4	D5	Overall
Allen et al. 2021	TF-CBT	TF-CBT	PTSS, depression, anxiety	+	+	+	+	!	!
Brown et al. 2020	TF-CBT	TAU/AT	PTSS, depression, anxiety, grief	!	+	+	+	!	!
Cohen et al. 1998, 2005	TF-CBT	TAU/AT	PTSS, depression, anxiety	+	+	+	+	!	!
Davidson et al. 2018	TF-CBT	TF-CBT	PTSS, depression	+	-	+	+	!	-
Diehle et al. 2015	TF-CBT	TAU/AT	PTSS, depression, anxiety	+	+	+	+	!	!
Dorsey et al. 2014	TF-CBT	TF-CBT	PTSS, depression	+	+	+	+	!	!
Dorsey et al. 2020	TF-CBT Group	TAU/AT	PTSS, grief	+	+	+	+	+	+
Goldbeck et al. 2016; Tutus et al. 2017	TF-CBT	WL	PTSS, depression, anxiety	+	+	+	+	+	+
Jensen et al. 2014, 2017	TF-CBT	TAU/AT	PTSS, depression, anxiety	+	+	+	+	+	+
King et al. 2000	TF-CBT	WL	PTSS, depression, anxiety	+	+	+	-	!	-
Langley et al. 2015	TF-CBT Group	WL	PTSS, depression, anxiety	+	+	+	!	!	!
Nixon et al. 2012, 2017	TF-CBT	TAU/AT	PTSS, depression, anxiety	+	+	+	+	!	!
O'Callaghan et al. 2013	TF-CBT Group	WL	PTSS	+	+	+	+	!	!
O'Callaghan et al. 2015	TF-CBT Group	TAU/AT	PTSS	+	+	+	+	!	!
Salloum et al. 2015	TF-CBT	SC-TF-CBT[a]	PTSS	+	+	+	+	!	!
Santiago et al. 2018	TF-CBT Group	WL	PTSS, depression, anxiety	+	+	+	!	!	!

+ Low risk
! Some concerns
- High risk

D1 Randomisation process
D2 Deviations from the intended interventions
D3 Missing outcome data
D4 Measurement of the outcome
D5 Selection of the reported result

Note. TF-CBT = Trauma Focused Cognitive Behavioral Therapy; TAU = Treatment As Usual; AT = Active treatment; WL = Wait-List; SC-TF-CBT = Stepped Care Trauma Focused Cognitive Behavioral Therapy; PTSS = Posttraumatic Stress Symptoms.

[a] SC-TF-CBT was excluded from analyses as some participants received TF-CBT while others did not.

Per-protocol

Unique ID	Experimental	Comparator	Outcome	D1	D2	D3	D4	D5	Overall
Cohen et al. 2004; Deblinger et al. 2006	TF-CBT	TAU/AT	PTSS, depression, anxiety	+	+	+	+	!	!
Deblinger et al. 1996, 1999	TF-CBT	TAU/AT	PTSS, depression, anxiety	+	+	+	+	!	!
Jaberghaderi et al. 2004	TF-CBT	TAU/AT	PTSS	+	-	+	+	!	-
McMullen et al. 2013	TF-CBT Group	WL	PTSS	+	+	+	+	+	+
Schottelkorb et al. 2012	TF-CBT	TAU/AT	PTSS	+	+	+	-	!	-

+ Low risk
! Some concerns
- High risk

D1 Randomisation process
D2 Deviations from the intended interventions
D3 Missing outcome data
D4 Measurement of the outcome
D5 Selection of the reported result

Note. TF-CBT = Trauma Focused Cognitive Behavioral Therapy; TAU = Treatment As Usual; AT = Active treatment; WL = Wait-List; PTSS = Posttraumatic Stress Symptoms.

Intention-to-treat

Unique ID	Experimental	Outcome	D2	D3	D4	D5	Overall
Cohen, Mannarino et al. 2004	TF-CBT	PTSS, depression, anxiety, grief	+	+	!	!	!
O'Donnel et al. 2014	TF-CBT Group	PTSS, depression, grief	+	+	!	!	!
Stewart et al. 2017	TF-CBT	PTSS, depression, anxiety	+	+	-	!	-
Unterhitzenberger et al. 2019	TF-CBT	PTSS, depression	+	+	!	!	!
Webb et al. 2014	TF-CBT	PTSS	+	+	-	-	-

+ Low risk
! Some concerns
- High risk

D2 Deviations from the intended interventions
D3 Missing outcome data
D4 Measurement of the outcome
D5 Selection of the reported result

Note. TF-CBT = Trauma Focused Cognitive Behavioral Therapy; PTSS = Posttraumatic Stress Symptoms.

Per-protocol

Unique ID	Experimental	Outcome	D2	D3	D4	D5	Overall
Amaya-Jackson et al. 2018	TF-CBT	PTSS, depression	+	!	!	!	!
Cohen et al. 2006	TF-CBT	PTSS, depression, anxiety, grief	+	!	!	!	!
Espeleta et al. 2021	TF-CBT	PTSS, depression	!	+	!	!	!
Kirsch et al. 2011	TF-CBT	PTSS	?	-	-	!	-
Konanur et al. 2015	TF-CBT	PTSS	+	-	!	-	-
Lang et al. 2015	TF-CBT	PTSS, depression	+	-	-	!	-
Murray et al. 2013	TF-CBT	PTSS	+	-	!	!	-
Stewart et al. 2020	TF-CBT	PTSS, depression	+	-	-	!	-

+ Low risk
! Some concerns
- High risk
? No information

D2 Deviations from the intended interventions
D3 Missing outcome data
D4 Measurement of the outcome
D5 Selection of the reported result

Note. TF-CBT = Trauma Focused Cognitive Behavioral Therapy; PTSS = Posttraumatic Stress Symptoms.

Table 30

Pre-FU I and FU II Within-Group Effect Sizes for PTSS, Depression and Anxiety With High Risk of Bias Studies.

Assessment point	Outcome	Sample	n	g	95% CI	SE	z	Q	I^2	Duval and Tweedie
Pre-FU I	PTSS	All studies	11	(1.70)<br>1.57[a]	(1.34 - 2.06)<br>1.21 - 1.93	(0.18)	(9.28***)	26.98**	62.94	2
	Depression	All studies	8	.82	.52 - 1.11	.15	5.45***	19.29**	63.71	0
	Anxiety	All studies	4	(.62[b])<br>.38[a,b]	(.37 - .88)<br>.17 - .59	(.13)	(4.79***)	5.00	40.01	2
Pre-FU II	PTSS	All studies	10	1.50	.95 - 2.06	.28	5.29***	113.67***	92.08	0
Pre-FU III	PTSS	All studies	14	(1.93)<br>1.68	(1.52 - 2.35)<br>1.26 - 2.09	(.21)	(9.15***)	124.27***	89.54	3

Note. High risk of bias studies included; n = number of included TF-CBT conditions. * $p < .05$; ** $p < .01$; *** $p < .001$

[a] Recalculated with imputed studies (trim and fill method according to Duval and Tweedie).

[b] Fixed model assumed due to non-significant Q-value.

Table 31

Within-Group and Between-Group Effect Sizes for Proxy-Reported PTSS, Depression and Anxiety With High Risk of Bias Studies.

Analysis	Assessment point	Outcome	n	g	95% CI	SE	z	Q	I^2	Duval and Tweedie
Within-group	Pre-Post	PTSS	28	1.01	.79 - 1.24	.12	8.84***	248.11***	89.12	0
		Depression	10	(.61)<br>.57[a]	(.44 - .77)<br>.39 - .74	(.09)	(7.18***)	18.39*	59.44	1
		Anxiety	3	.52[b]	.34 - .71	.09	5.55***	.08	0	0
	Pre-FU II	PTSS	3	.88	.30 - .1.46	.09	2.96**	12.86	84.45	0
Between-group	Post	PTSS	9	.46	.09 - .83	.19	2.45*	57.00***	85.97	0

Note. High risk of bias studies included; n = number of included comparisons; PTSS = Posttraumatic Stress Symptoms.

* $p < .05$; ** $p < .01$; *** $p < .001$

[a] Recalculated with imputed studies (trim and fill method according to Duval and Tweedie).

[b] Fixed model assumed due to non-significant Q-value.

Tabel 32

Criteria for Efficacy and Effectiveness trials

Criterion	Efficacy	Effectiveness
Title	Efficacy trial…	Effectiveness trial…
Setting	University-affiliated clinic/highly controlled setting	dissemination and implementation in the 'real world'/less constraint settings (schools, primary mental health care, multiple clinics with different structures)
Inclusion criteria	Stringent (e.g. only single trauma, full-blown PTSD, no comorbidities, prerandomization run-in periods to exclude placebo-responders etc.)	Inclusive (multiple traumas, only few symptoms required, different patient characteristics etc.)
Statistical analysis	Completer analysis typical of efficacy	Intention-to-treat typical of effectiveness
Adaptations	None or only in such a way that they can be systematically evaluated (e.g. adding components)	To make intervention more acceptable to patient group (e.g. considering culturally important aspects, moving component order, spending more time on specific components)
Therapists	Highly trained, standardized training	Less training or variation in training
Caregivers	Participation required	Not required or only if available
Control Condition	Waitlist not allowing for other treatments, if active, also manualized and highly controlled, more than 1 TF-CBT condition (dismantling studies)	Everything that can be considered treatment as usual and is not highly controlled
